# T_H_17–Associated Polyamine Metabolism‐Guided Nanozyme Promotes Alveolar Bone Repair in Periodontitis

**DOI:** 10.1002/advs.77247

**Published:** 2026-08-18

**Authors:** Cheng Zhu, Tiancheng Li, Yu Jin, Yixin Li, Qicheng Liu, Runzhe Fang, Luyao Qu, Xinyue Tang, Ruomei Li, Xiaoyue Zhao, Jin Hao, Bing Fang, Lunguo Xia

**Affiliations:** ^1^ Department of Orthodontics College of Stomatology National Center for Stomatology National Clinical Research Center for Oral Diseases Shanghai Ninth People's Hospital Shanghai Jiao Tong University School of Medicine Shanghai Jiao Tong University Shanghai Key Laboratory of Stomatology Shanghai Research Institute of Stomatology Shanghai China; ^2^ School of Food Science and Pharmaceutical Engineering Nanjing Normal University Nanjing China

**Keywords:** metal‐organic frameworks, periodontitis, ROS scavenging, T_H_17 cells

## Abstract

Periodontitis is a chronic inflammatory disease characterized by persistent inflammation and limited repair at sites of alveolar bone loss. Although excessive reactive oxygen species (ROS) are recognized drivers of periodontal tissue damage, oxidative stress alone does not fully explain the persistence of inflammation and the failure of regeneration. The immune and metabolic processes that cooperate with oxidative stress in sustaining this disease‐supporting microenvironment remain incompletely defined. In this study, a pronounced T_H_17‐skewed CD4^+^ T cell response is identified in experimental periodontitis, and enrichment of polyamine pathway activation is observed during T_H_17 differentiation, suggesting a candidate immunometabolic axis for intervention. Guided by this observation, difluoromethylornithine‐loaded UiO‐66(Ce)‐Mn (DFMO@Ui‐Mn) is developed as a nanozyme platform that integrates the cascade ROS‐scavenging activity of UiO‐66(Ce)‐Mn with DFMO delivery for inhibition of polyamine metabolism. DFMO@Ui‐Mn reduces T_H_17 polarization in vitro, alters polyamine‐related metabolic profiles, attenuates inflammatory responses in vivo, lowers the T_H_17/Treg ratio, improves osteogenic readouts in a conditioned‐medium model, and promotes alveolar bone repair in ligature‐induced periodontitis. These findings support a therapeutic strategy that combines redox control with immunometabolic intervention to improve the periodontal microenvironment under inflammatory conditions.

## Graphical abstract

A pronounced T_H_17‐skewed immune response is identified in experimental periodontitis, and enrichment of the polyamine pathway is observed during T_H_17 differentiation, suggesting its potential as an immunometabolic target. Inspired by these findings, DFMO@Ui‐Mn is developed as a therapeutic platform combining cascade ROS scavenging with immunometabolic modulation to suppress T_H_17 polarization, reduce inflammation, and promote alveolar bone repair in periodontitis.

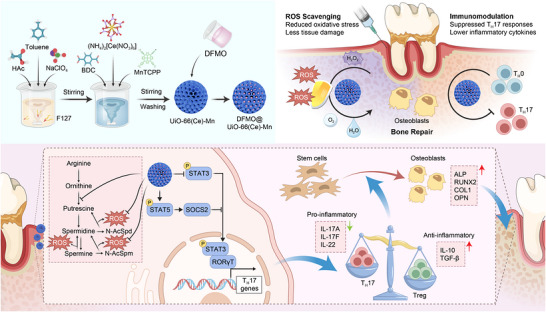

## 1 Introduction

Periodontitis is a prevalent chronic inflammatory disease affecting nearly 60% of the global population [1]. It is a major cause of tooth loss and is associated with a higher risk of systemic conditions such as myocardial infarction and Alzheimer's disease [2]. Although conventional mechanical therapy reduces bacterial plaque, achieving regeneration at sites with established inflammatory bone loss remains a clinical challenge [3]. This limitation suggests that the key barrier to periodontal tissue regeneration is closely associated with the persistent inflammatory pathological microenvironment that remains even after treatment. Among the pathological factors involved, oxidative stress is a recognized driver of periodontal bone resorption, and reactive oxygen species (ROS)‐scavenging strategies have shown protective effects in experimental models [4]. However, antioxidant intervention alone is unlikely to fully restore a repair‐supportive microenvironment, because oxidative stress does not act in isolation. Additional disease‐sustaining factors that cooperate with oxidative stress need to be defined and therapeutically addressed [5].

The pathological microenvironment of periodontitis, including non‐resolving inflammation, microbial dysbiosis, and oxidative stress, sustains and amplifies immune dysregulation [6]. Once established, this immune imbalance may persist even after standard therapy because altered immune cell states can impair microbial clearance, while established inflammatory networks may not be readily normalized, thereby impairing periodontal tissue healing [7, 8]. Central to this imbalance are CD4^+^ T cells, particularly the T helper cell 17 (T_H_17) subset, which drives bone destruction by inducing RANKL expression [9]. Elevated T_H_17 frequencies and IL‐17 levels correlate closely with the severity of alveolar bone loss [10, 11, 12, 13]. In addition, IL‐17 promotes neutrophil recruitment and amplifies ROS burden [14, 15], forming a self‐reinforcing pathogenic loop that sustains tissue damage. These findings highlight the need for immunomodulatory strategies, particularly those aimed at restraining T_H_17‐associated responses, to limit inflammatory bone loss and improve the tissue environment for repair [16].

Beyond cytokine signaling, the pathogenic activity of T_H_17 cells is increasingly recognized to depend on metabolic programming [17, 18]. To support rapid expansion and pro‐inflammatory secretion, T_H_17 cells undergo profound metabolic shifts. Emerging evidence indicates that amino acid‐associated metabolic pathways shape T_H_17 differentiation, persistence, and effector function [19]. Amino acid transporters have been shown to play essential roles in providing substrates and regulating T cell differentiation [20]. These metabolic adaptations contribute to T_H_17 differentiation and effector function. However, in periodontitis, the metabolic programs associated with T_H_17 responses remain incompletely defined. Whether targeting such programs can attenuate immune dysregulation and improve the periodontal tissue environment remains an important question.

Given the reciprocal reinforcement between oxidative stress and T_H_17‐driven immune pathology, an effective intervention should ideally modulate immunometabolism while providing local ROS buffering. In this context, metal‐organic frameworks (MOFs) offer distinct advantages, as their metal‐ligand architectures endow catalytic antioxidant activity and their structural tunability supports the rational integration of therapeutic functions [21, 22]. Among them, UiO‐66(Ce)‐based Mn‐functionalized MOFs are particularly suitable, as the exposed Ce‐O clusters and Mn catalytic sites support cascade ROS scavenging, and the porous framework allows efficient encapsulation of immunometabolic agents [23, 24, 25]. These features make them promising therapeutic platforms for periodontitis‐related inflammatory bone resorption.

**SCHEME 1:**
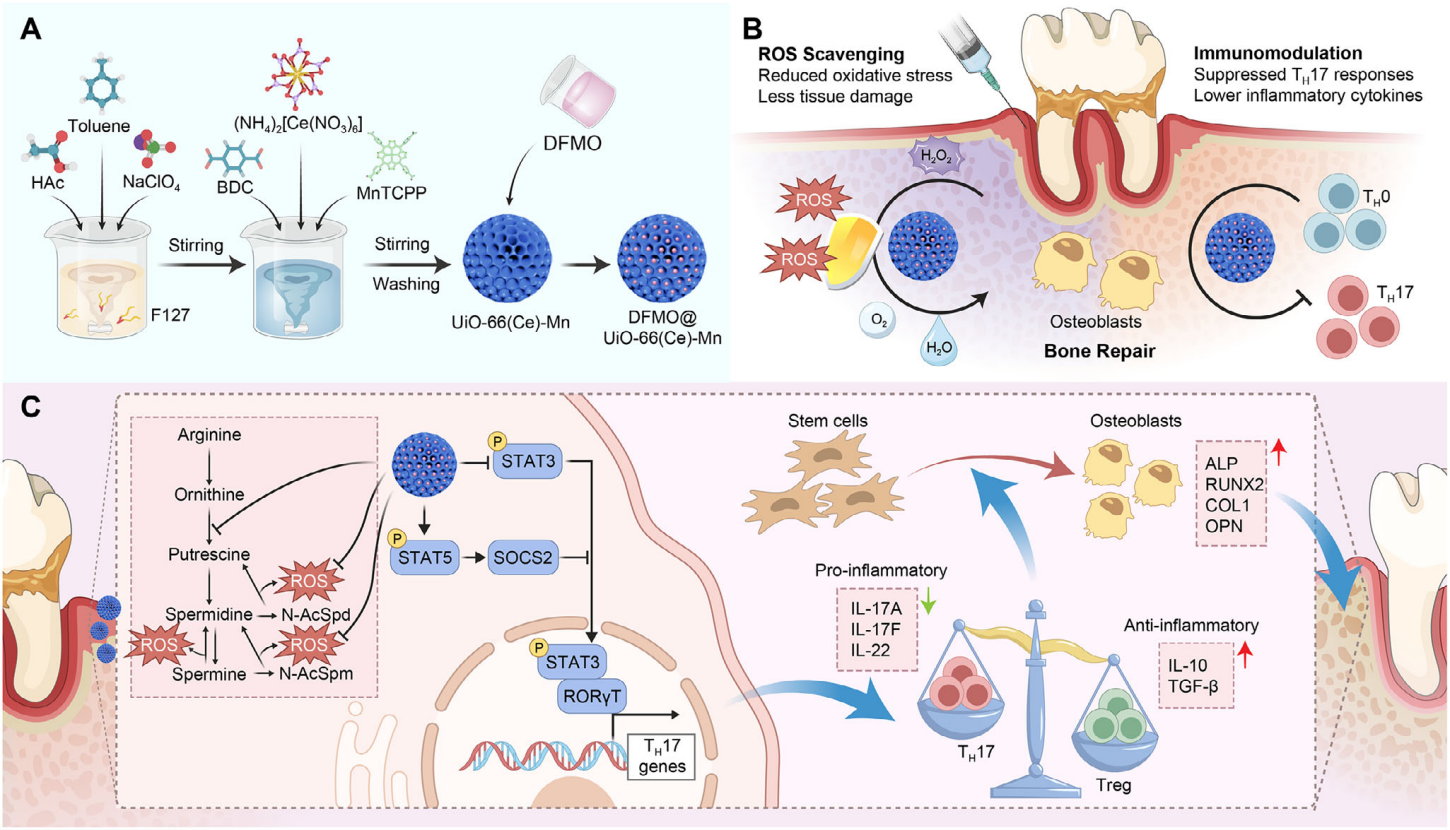
(A) Schematic synthesis of DFMO@UiO‐66(Ce)‐Mn. (B) The dual‐functional nanozyme provides cascade ROS scavenging and immunomodulation to reduce oxidative stress and suppress T_H_17‐driven inflammation, supporting bone repair. (C) Mechanistic illustration showing that DFMO inhibits polyamine metabolism, while UiO‐66(Ce)‐Mn alleviates ROS, collectively reducing T_H_17 polarization through altered STAT3/STAT5 signaling and SOCS2 upregulation, promoting alveolar bone repair.

Here, we profiled CD4^+^ T cell responses in experimental periodontitis and characterized metabolic features associated with in vitro T_H_17 differentiation, identifying polyamine pathway activation as a candidate immunometabolic feature linked to T_H_17 responses. On this basis, we developed difluoromethylornithine‐loaded UiO‐66(Ce)‐Mn, denoted as DFMO@UiO‐66(Ce)‐Mn and abbreviated as DFMO@Ui‐Mn, as a nanozyme‐based therapeutic system for combined redox and metabolic intervention (Scheme 1). In this system, the UiO‐66(Ce)‐Mn (Ui‐Mn) carrier provides rapid ROS‐scavenging activity through synergistic catalytic activity of Ce clusters and Mn‐porphyrin centers, while DFMO is released to inhibit ornithine decarboxylase (ODC), the rate‐limiting enzyme in polyamine biosynthesis. Compared with ROS regulation alone, the combined formulation more effectively modulated T_H_17‐associated responses and improved inflammatory and bone‐loss readouts in this model. These findings support polyamine metabolism as a therapeutically relevant immunometabolic axis in periodontitis and suggest that combining redox control with immune modulation may improve the periodontal microenvironment.

## 2 Results

### 2.1 Experimental Periodontitis Is Associated With an Enhanced T_H_17 Response, and T_H_17 Differentiation Is Accompanied by Polyamine Pathway Activation

**FIGURE 1:**
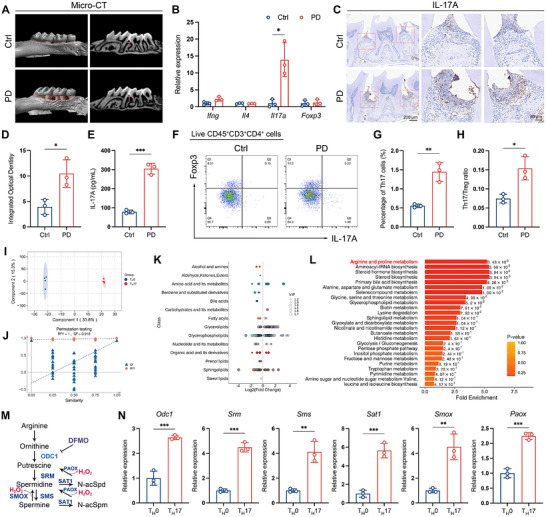
T_H_17 responses are elevated in periodontitis and are associated with polyamine pathway activation during T_H_17 differentiation. (A) Representative micro‐CT images of mouse maxillae from control (Ctrl) and ligature‐induced periodontitis (PD) groups. (B) RT‐qPCR analysis of *Ifng*, *Il4*, *Il17a*, and *Foxp3* mRNA in gingival tissues (*n* = 3). (C) Representative IHC staining of IL‐17A in maxillary periodontal tissues (boxed regions shown at higher magnification). (D) Quantification of IL‐17A IHC signal between the first and second molars (*n* = 3). (E) IL‐17A levels in gingival tissues surrounding the maxillary second molar measured by ELISA (*n* = 3). (F) Representative flow cytometry plots of IL‐17A and Foxp3 within live CD45^+^CD3^+^CD4^+^ lymphocytes isolated from cervical lymph nodes. (G) Frequency of T_H_17 cells in cervical lymph nodes (*n* = 3). (H) T_H_17/Treg ratio in cervical lymph nodes (*n* = 3). (I) OPLS‐DA score plot of untargeted metabolomics profiles (*n* = 4). (J) Permutation test for the OPLS‐DA model. (K) Bubble plot of differential metabolites by class. (L) MSEA showing the top enriched pathways. (M) Schematic of polyamine synthesis and catabolism. (N) RT‐qPCR of polyamine pathway‐related genes in T_H_0 and T_H_17 cells (*n* = 3). Data are presented as mean ± standard deviation and were analyzed using Student's *t*‐test. *p < 0.05, **p < 0.01, ***p < 0.001.

To examine the CD4^+^ T cell‐associated response in periodontitis, a ligature‐induced periodontitis model was established at the maxillary second molar. Micro‐CT analysis confirmed alveolar bone loss in the ligated group (Figure 1A and Figure S1). RT‐qPCR analysis of gingival tissues showed that, among representative helper T cell‐associated markers examined, *Il17a* exhibited the most prominent upregulation in periodontitis (Figure 1B). Immunohistochemistry (IHC) revealed increased IL‐17A‐positive signals in periodontal tissues surrounding the second molar (Figure 1C,D), consistent with higher IL‐17A levels detected in gingival tissue extracts by ELISA (Figure 1E). Flow cytometry revealed an increased frequency of T_H_17 cells and an elevated T_H_17/Treg ratio in cervical lymph nodes from mice with periodontitis (Figure 1F–H). Together, these results indicate that experimental periodontitis is accompanied by a pronounced T_H_17‐biased immune response.

To characterize metabolic features associated with T_H_17 differentiation, untargeted metabolomics profiling was performed by comparing T_H_17‐polarized CD4^+^ T cells with T_H_0 controls. The score plots of orthogonal partial least squares‐discriminant analysis (OPLS‐DA) and principal component analysis (PCA) showed distinct separation between the two groups (Figure 1I and Figure S2A), and permutation testing supported the validity of the model (Q^2^ = 0.918, *p* < 0.005; Figure 1J). Differential metabolite patterns were visualized by the volcano plot and hierarchical clustering heatmap (Figure S2B,C). In the differential‐metabolite scatter plot, larger absolute log_2_ fold changes were frequently observed among amino acid‐related metabolites and glycerophospholipids (Figure 1K). Metabolite set enrichment analysis (MSEA) highlighted arginine and proline metabolism as the top enriched pathway (Figure 1L), suggesting substantial remodeling of arginine‐related metabolism in T_H_17 cells. Given that polyamine biosynthesis is a major downstream branch of arginine metabolism and has been implicated in immune regulation [26], we next examined this pathway as a candidate metabolic node underlying the T_H_17‐associated response (Figure 1M). The expression of key enzymes involved in polyamine metabolism was then further assessed by RT‐qPCR. RT‐qPCR analysis showed that T_H_17 cells expressed higher levels of polyamine biosynthesis‐related genes (*Odc1*, *Sms* and *Srm*) together with polyamine catabolism‐related genes (*Sat1*, *Smox* and *Paox*) than T_H_0 controls (Figure 1N). Consistently, representative immunoblotting suggested increased ODC1 protein expression in T_H_17 cells compared with T_H_0 cells (Figure S3). To functionally test the relevance of ODC activity, treatment with DFMO, an irreversible ODC inhibitor, reduced IL‐17A^+^ T_H_17 polarization. This inhibitory effect was rescued by supplementation with putrescine, the direct polyamine product generated from ornithine by ODC, whereas putrescine alone did not further enhance T_H_17 polarization under the same induction conditions (Figure S4). In summary, experimental periodontitis exhibits a pronounced T_H_17‐biased immune response in vivo, while in vitro T_H_17 differentiation was accompanied by enrichment of arginine‐related metabolic programs and increased expression of polyamine metabolism genes.

### 2.2 Construction and Characterization of DFMO@UiO‐66(Ce)‐Mn

**FIGURE 2:**
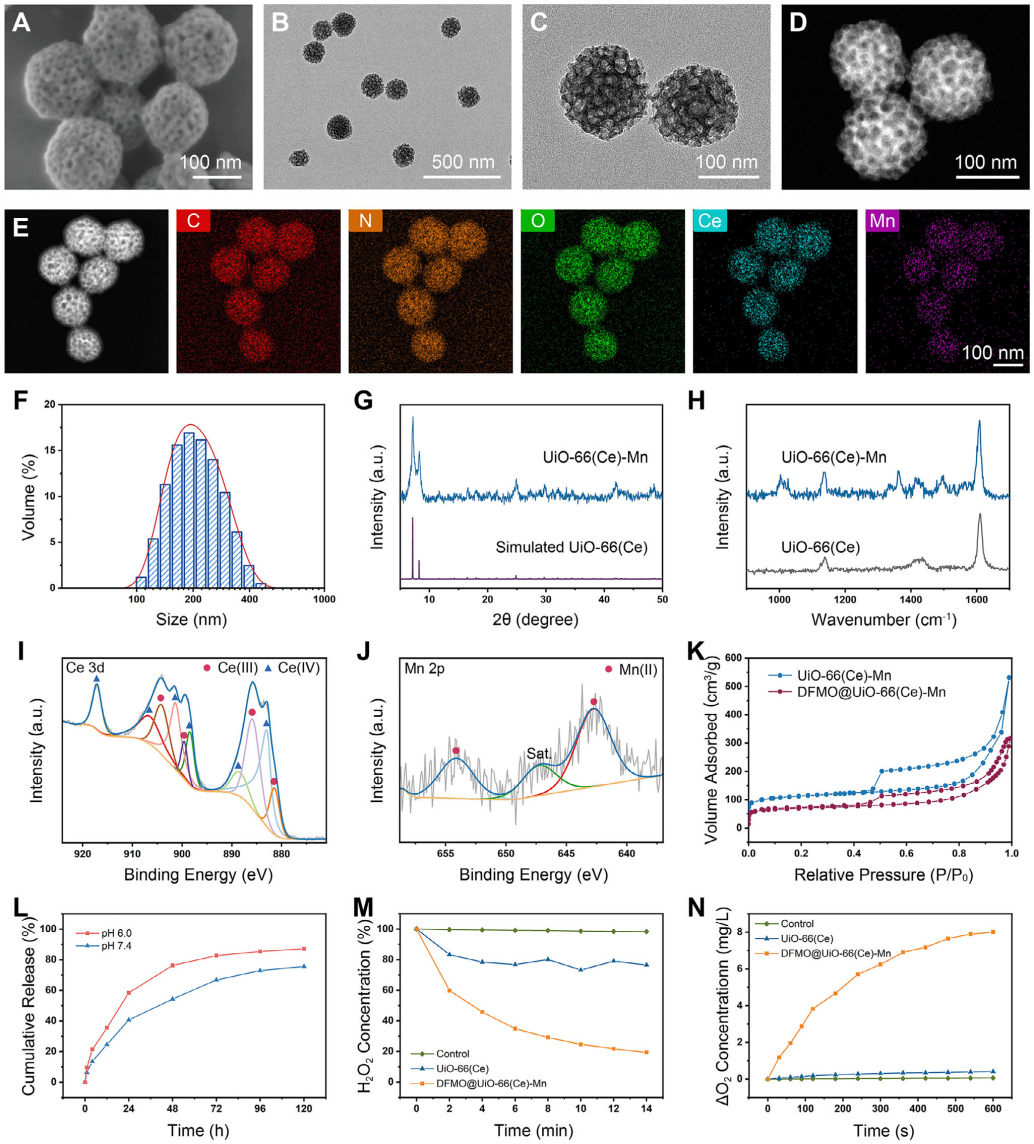
Characterization and functional properties of DFMO@UiO‐66(Ce)‐Mn. (A) Representative SEM image of UiO‐66(Ce)‐Mn nanoparticles. (B,C) Representative TEM images of UiO‐66(Ce)‐Mn nanoparticles. (D) HAADF‐STEM image of UiO‐66(Ce)‐Mn. (E) Elemental mapping showing the distribution of C, N, O, Ce and Mn in UiO‐66(Ce)‐Mn. (F) Hydrodynamic size distribution of UiO‐66(Ce)‐Mn measured by DLS. (G) XRD patterns of UiO‐66(Ce)‐Mn and simulated UiO‐66(Ce). (H) Raman spectra of UiO‐66(Ce) and UiO‐66(Ce)‐Mn. (I) XPS spectrum of Ce 3d for UiO‐66(Ce)‐Mn. (J) XPS spectrum of Mn 2p for UiO‐66(Ce)‐Mn. (K) N_2_ adsorption–desorption isotherms of UiO‐66(Ce)‐Mn and DFMO@UiO‐66(Ce)‐Mn. (L) DFMO release profiles of DFMO@UiO‐66(Ce)‐Mn at pH 7.4 and pH 6.0. (M) Dynamic H_2_O_2_ elimination activity. (N) Dynamic O_2_ generation from H_2_O_2_ decomposition.

To couple T_H_17 immunometabolic blockade with antioxidative protection, DFMO@UiO‐66(Ce)‐Mn was prepared by loading DFMO into UiO‐66(Ce)‐Mn, in which MnTCPP was incorporated as a porphyrinic ligand. UiO‐66(Ce)‐Mn was synthesized through an F127‐assisted micellar route in the aqueous phase, where stable spherical micelles enabled the controlled growth of UiO‐66(Ce) under Hofmeister ion ClO_4_^−^ mediation [27]. Toluene was introduced as a swelling agent to tune mesoporosity. Meanwhile, MnTCPP served as a multidentate porphyrinic ligand embedded into the framework, providing catalase‐like activity that complements the Ce‐node‐derived SOD‐like redox catalysis. To balance particle morphology and catalytic performance, the feed ratio of MnTCPP to BDC was optimized to 0.05. Morphologically, SEM revealed uniform spherical nanoparticles with a porous, roughened surface (Figure 2A). TEM further confirmed well‐dispersed particles with an average diameter of around 150 nm (Figure 2B,C), while HAADF‐STEM imaging showed distinct contrast consistent with a porous framework architecture (Figure 2D). Elemental mapping of individual nanoparticles demonstrated homogeneous distributions of C, N, O, Ce, and Mn (Figure 2E), supporting the successful incorporation of Mn‐porphyrins into the UiO‐66(Ce) framework. Dynamic light scattering (DLS) gave a larger hydrodynamic diameter of around 216 nm (Figure 2F), which is expected relative to TEM due to solvation layers and potential interparticle association in aqueous dispersion.

Structural integrity of the UiO‐66(Ce) framework after MnTCPP incorporation was supported by XRD, which showed preserved diffraction features consistent with UiO‐66(Ce) crystallinity (Figure 2G). In the Raman window of 1000–1650 cm^−1^, dominated by aromatic ring and linker‐associated vibrations, UiO‐66(Ce)‐Mn retained the main framework‐associated bands while exhibiting additional porphyrin‐related features compared with UiO‐66(Ce), consistent with successful introduction of the TCPP macrocycle (Figure 2H). XPS further verified the redox‐active composition: Ce 3d spectra indicated coexistence of Ce(III) and Ce(IV) species in UiO‐66(Ce)‐Mn (Figure 2I), while Mn 2p spectra confirmed the presence of Mn species (Figure 2J), together supporting the intended dual redox centers.

The N_2_ adsorption–desorption isotherms of UiO‐66(Ce)‐Mn and DFMO@UiO‐66(Ce)‐Mn exhibited type IV characteristics with H4‐type hysteresis loops (Figure 2K), consistent with the coexistence of micro‐ and mesoporous features. After DFMO loading, the Brunauer–Emmett–Teller (BET) surface area decreased from 416.6 to 262.5 m^2^/g, while the total pore volume decreased from 0.82 to 0.49 cm^3^/g (Table S1). The reduced surface area and pore volume, together with the retained isotherm characteristics, supported the occupation of the porous structure by DFMO without substantial framework collapse. DFMO@UiO‐66(Ce)‐Mn exhibited an encapsulation efficiency of 23.1% and a drug loading content of 31.6 wt.%. To further evaluate the particle stability under physiologically relevant conditions, DFMO@UiO‐66(Ce)‐Mn was incubated in HEPES‐buffered saline (HBS, pH 7.4), HBS (pH 6.5), and HBS (pH 7.4) containing 10% fetal bovine serum (FBS) for 3 days. The nanoparticles maintained nanoscale hydrodynamic size distributions with mildly negative zeta potentials (−6.18, −9.29, and −5.03 mV, respectively), and TEM images showed that their spherical morphology was largely preserved in these media (Figure S5). The release behavior of DFMO@UiO‐66(Ce)‐Mn was then evaluated, showing faster DFMO release under mildly acidic conditions (pH 6.0) than at physiological pH (7.4), with a higher cumulative release over 120 h (Figure 2L). This release profile may favor early metabolic intervention and sustained local drug availability. To evaluate catalase (CAT)‐like activity, H_2_O_2_ decomposition was monitored over time. While UiO‐66(Ce) adsorbed H_2_O_2_, it showed little catalytic consumption of H_2_O_2_; after reaching an adsorption‐desorption equilibrium within the porous channels, the signal remained nearly unchanged. DFMO@UiO‐66(Ce)‐Mn markedly accelerated H_2_O_2_ depletion (Figure 2M), indicating enhanced CAT‐mimic activity after Mn‐porphyrin incorporation. In parallel, O_2_ generation in H_2_O_2_ solution was substantially increased for DFMO@UiO‐66(Ce)‐Mn, while UiO‐66(Ce) produced only a minimal O_2_ increase over the same time window (Figure 2N), further supporting the boosted catalytic conversion of H_2_O_2_ to O_2_ by the MnTCPP‐containing framework.

### 2.3 DFMO@Ui‐Mn Attenuates T_H_17 Polarization and Is Accompanied by Polyamine‐related Metabolic Remodeling

To support subsequent biological studies, the in vitro biosafety of DFMO@UiO‐66(Ce)‐Mn (DFMO@Ui‐Mn) was evaluated in CD4^+^ T cells. DFMO@Ui‐Mn showed no obvious cytotoxicity at concentrations up to 5 µg/mL, as assessed by CCK‐8, Zombie viability dye staining, and Annexin V/PI flow cytometry (Figure S6). At higher concentrations (≥25 µg/mL), a dose‐dependent reduction in cell viability accompanied by increased apoptosis was observed (Figure S6). Therefore, 5 µg/mL was selected as a safe working concentration for subsequent experiments.

**FIGURE 3:**
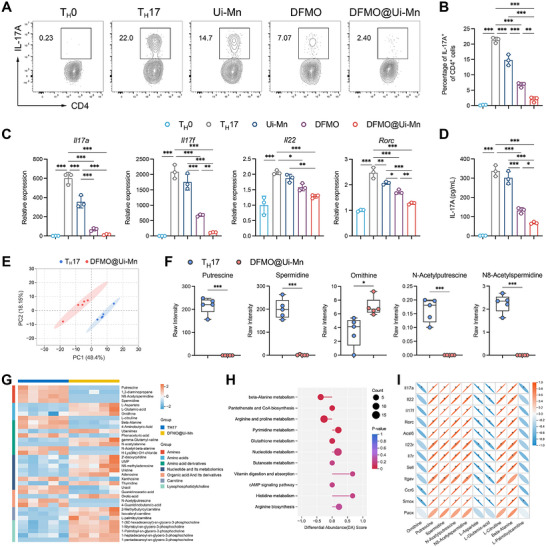
DFMO@Ui‐Mn suppresses T_H_17 polarization and is associated with polyamine‐related metabolic remodeling in vitro. (A) Representative flow cytometry plots showing IL‐17A expression in CD4^+^ T cells cultured in vitro under the indicated conditions. (B) Quantification of IL‐17A^+^ cells among CD4^+^ T cells (*n* = 3). (C) RT‐qPCR analysis of T_H_17‐associated genes (*Il17a*, *Il17f*, *Il22*, and *Rorc*) in the indicated groups (*n* = 3). (D) ELISA measurement of IL‐17A in culture supernatants of CD4^+^ T cells (*n* = 3). (E) PCA score plot of targeted metabolomics profiles comparing T_H_17 and DFMO@Ui‐Mn–treated groups (*n* = 5). (F) Raw intensities of representative polyamine‐related metabolites (*n* = 5). (G) Heatmap of differential metabolites between T_H_17 and DFMO@Ui‐Mn groups, annotated by metabolite class (*n* = 5). (H) KEGG pathway analysis of differential metabolites shown as differential abundance score. (I) Pearson correlation matrix integrating transcriptomic and metabolomic features, showing correlations between T_H_17‐related genes, polyamine‐oxidation genes, and representative metabolites. Data in panels B–D are presented as mean ± standard deviation and were analyzed using one‐way ANOVA followed by Tukey's multiple‐comparisons test. Data in panel F are presented as box‐and‐whisker plots with the whiskers extending from the minimum to the maximum and were analyzed using Student's *t*‐test. *p < 0.05, **p < 0.01, ***p < 0.001.

To evaluate the impact of the materials on T_H_17 differentiation, naïve CD4^+^ T cells were isolated and cultured under T_H_17‐polarizing conditions, followed by treatment with Ui‐Mn, DFMO, or DFMO@Ui‐Mn. Flow cytometry showed that T_H_17 polarization markedly increased the frequency of IL‐17A^+^ cells relative to the T_H_0 control (Figure 3A,B). Ui‐Mn alone led to a modest reduction in IL‐17A^+^ cells. DFMO further decreased the proportion of IL‐17A^+^ cells, whereas DFMO@Ui‐Mn produced the most pronounced suppression among all groups (Figure 3A,B). Accordingly, RT‐qPCR analysis demonstrated marked induction of T_H_17‐associated transcripts (*Il17a*, *Il17f*, *Il22*, and *Rorc*) in the T_H_17 group, all of which were significantly downregulated by DFMO@Ui‐Mn (Figure 3C). Reduced T_H_17 response was further supported by ELISA, showing decreased IL‐17A secretion in culture supernatants following DFMO@Ui‐Mn treatment (Figure 3D).

To evaluate the metabolic impact of DFMO@Ui‐Mn on T_H_17 cells, targeted metabolomics profiling was performed. PCA showed a clear separation between the T_H_17 and DFMO@Ui‐Mn groups (Figure 3E). DFMO@Ui‐Mn markedly reduced levels of polyamine‐related metabolites, including putrescine, spermidine and their acetylated derivatives such as N‐acetylputrescine and N8‐acetylspermidine (Figure 3F). The class‐annotated heatmap further indicated that differential metabolites were predominantly enriched in amines and amino acids/derivatives and extended to nucleotide‐related metabolites (Figure 3G). In parallel, lipid‐associated changes were also observed, characterized by increased levels of several carnitine species linked to fatty acid oxidation and elevated lysophospholipids (Figure 3G). Consistently, Kyoto Encyclopedia of Genes and Genomes (KEGG) pathway analysis ranked arginine and proline metabolism among the top enriched pathways, together with multiple amino acid‐related pathways (Figure 3H).

To interrogate the relationship between polyamine metabolism and T_H_17 programming, we performed an integrated analysis of targeted metabolomics and transcriptomic data. Pearson correlation analysis revealed a tight association between the T_H_17 transcriptional program and the polyamine module (Figure 3I). Key T_H_17‐related genes (*Il17a*, *Il17f*, *Il22*, *Rorc*, *Il23r*, and *Il7r*), together with activation/trafficking markers (*Sell*, *Itgav*, and *Ccr6*), showed consistent positive correlations with polyamine metabolites and their acetylated derivatives, including putrescine, spermidine, N‐acetylputrescine, and N8‐acetylspermidine. Notably, the polyamine oxidation‐related genes *Smox* and *Paox* exhibited correlation patterns aligned with these polyamine species (Figure 3I). Beyond the polyamine module, additional metabolites also exhibited measurable associations with T_H_17‐related genes, including several amino acids (e.g., L‐aspartate and L‐glutamate) and a lipid‐related feature (L‐palmitoylcarnitine). Together, these correlation patterns suggest that T_H_17‐related transcriptional features covary with metabolic changes.

### 2.4 DFMO@Ui‐Mn Partially Restores Osteogenic Differentiation of BMSCs Impaired by T_H_17‐Conditioned Medium

**FIGURE 4:**
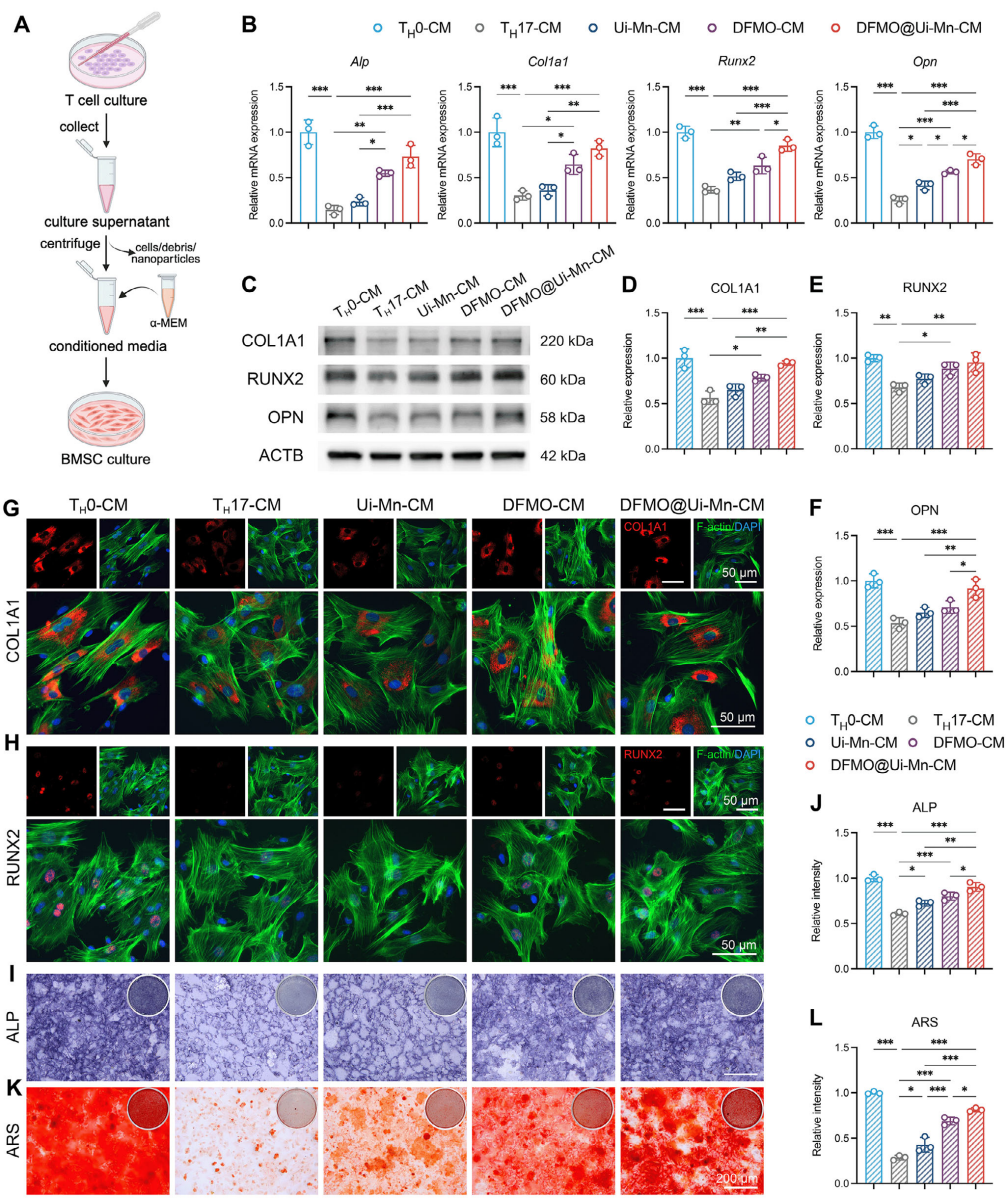
DFMO@Ui‐Mn alleviates T_H_17‐impaired BMSC osteogenesis. (A) Schematic of T cell–conditioned medium (CM) preparation and its application to BMSCs. (B) RT‐qPCR analysis of osteogenesis‐related genes in BMSCs cultured with the indicated CM (*n* = 3). (C) Representative immunoblots of osteogenic markers (COL1A1, RUNX2, and OPN) and quantitative analysis of (D) COL1A1, (E) RUNX2, and (F) OPN normalized to ACTB (*n* = 3). Representative immunofluorescence images of (G) COL1A1 and (H) RUNX2 in BMSCs cultured with the indicated CM; F‐actin and nuclei were counterstained with phalloidin and DAPI. (I) ALP staining and (J) quantification (*n* = 3). (K) ARS staining for mineral deposition and (L) quantification (*n* = 3). Data are presented as mean ± standard deviation and were analyzed using one‐way ANOVA followed by Tukey's multiple‐comparisons test. *p < 0.05, **p < 0.01, ***p < 0.001.

Osteogenesis is regulated by the local immune microenvironment [9]. To assess how the T cell‐mediated microenvironment influences osteogenesis, conditioned medium (CM) was collected from in vitro CD4^+^ T cell cultures and applied to mouse bone marrow mesenchymal stem cells (BMSCs) (Figure 4A). Compared with T_H_0‐CM, T_H_17‐CM markedly reduced the expression of osteogenesis‐related genes (*Alp*, *Col1a1*, *Runx2*, *Opn*) in BMSCs, whereas CM from DFMO@Ui‐Mn‐treated T cells restored their expression (Figure 4B), consistent with the corresponding decrease in T_H_17 polarization and IL‐17A secretion. Western blotting (Figure 4C–F) and immunofluorescence (Figure 4G,H) further corroborated these findings at the protein level for COL1A1, RUNX2, and OPN. Functionally, ALP staining at day 7 (Figure 4I,J) and Alizarin Red S (ARS) staining at day 14 (Figure 4K,L) indicated that DFMO@Ui‐Mn effectively rescued the osteogenic activity and mineralization impaired by the T_H_17 milieu. Importantly, control experiments showed that adding free DFMO or MnCl_2_ to T_H_17‐CM did not reproduce the osteogenic rescue achieved by CM from DFMO@Ui‐Mn–treated T_H_17 cultures (Figure S7). ALP/ARS staining and RUNX2/OPN expression indicate that this osteogenic rescue is primarily attributed to DFMO@Ui‐Mn‐mediated remodeling of the T cell secretome, rather than a direct pharmacological effect of DFMO or Mn on BMSCs. Overall, these results suggest that DFMO@Ui‐Mn alters the T cell secretory profile in a manner associated with improved osteogenic differentiation of BMSCs.

### 2.5 DFMO@Ui‐Mn is Associated With Altered STAT3/STAT5 Signaling and SOCS2 Upregulation

**FIGURE 5:**
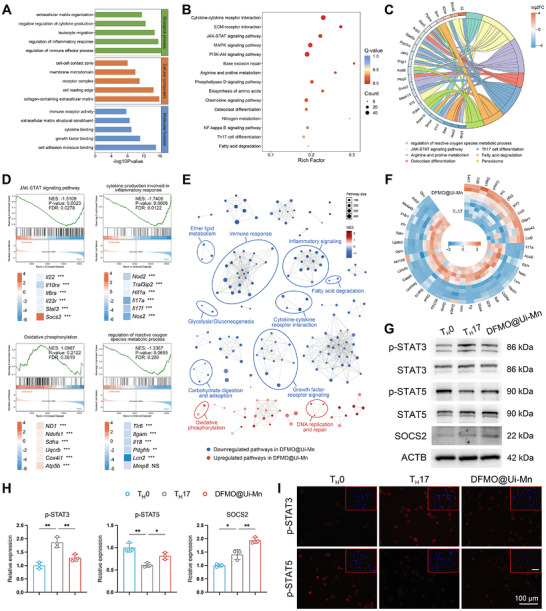
Transcriptomics reveals inflammatory suppression and metabolic remodeling by DFMO@Ui‐Mn. (A) GO enrichment of DEGs between DFMO@Ui‐Mn–treated and untreated T_H_17 cells, shown as representative terms from Biological Process, Cellular Component, and Molecular Function. (B) KEGG pathway enrichment analysis of DEGs of DFMO@Ui‐Mn vs T_H_17. (C) Chord diagram linking selected enriched pathways/processes to their contributing genes (colored by log_2_ fold change). (D) GSEA of selected terms, with corresponding gene heatmaps. (E) Network‐based clustering of GSEA‐KEGG enriched pathways to identify coordinated functional modules altered by DFMO@Ui‐Mn; pathways downregulated and upregulated in DFMO@Ui‐Mn relative to T_H_17 are shown in blue and red, respectively. (F) Heatmap of T_H_17‐associated inflammatory signature genes across groups (*n* = 5). (G) Representative immunoblots of p‐STAT3, STAT3, p‐STAT5, STAT5, and SOCS2 in T_H_0, T_H_17, and DFMO@Ui‐Mn groups, and (H) quantification of p‐STAT3, p‐STAT5, and SOCS2 normalized to ACTB (*n* = 3). (I) Immunofluorescence staining of p‐STAT3 and p‐STAT5 in T_H_0, T_H_17, and DFMO@Ui‐Mn groups. Data are presented as mean ± standard deviation and were analyzed using one‐way ANOVA followed by Tukey's multiple‐comparisons test. NS, not significant; *p < 0.05, **p < 0.01, ***p < 0.001.

To elucidate the immunomodulatory mechanisms of DFMO@Ui‐Mn, we performed RNA sequencing followed by functional enrichment analyses. Transcriptomic profiling showed clear separation between T_H_17 and DFMO@Ui‐Mn‐treated T_H_17 samples, with distinct differentially expressed gene (DEG) patterns visualized by PCA, volcano plot, and hierarchical clustering (Figure S8). Gene Ontology (GO) analysis revealed that dominant biological processes were related to inflammatory responses and cytokine production, with cellular components associated with receptor complexes and molecular functions focused on cytokine binding (Figure 5A). KEGG analysis identified enrichment in inflammation‐related pathways, including JAK–STAT and PI3K–Akt, accompanied by metabolic modules and oxidative stress processes (Figure 5B). Notably, JAK–STAT emerged as a central axis linking cytokine cues to T_H_17 programs.

The chord diagram visualized coordinated changes in T_H_17 immune programs together with metabolic and oxidative regulation in response to DFMO@Ui‐Mn (Figure 5C). Gene Set Enrichment Analysis (GSEA) showed negative enrichment of JAK–STAT signaling and inflammatory cytokine production signatures (Figure 5D). Oxidative phosphorylation was enriched, whereas ROS‐metabolic signatures were reduced, consistent with the antioxidative activity of the Ui‐Mn nanozyme component. Pathway‐network analysis indicated suppression of tightly connected immune modules with concomitant changes in metabolic and redox nodes (Figure 5E). Moreover, the heatmap confirmed broad downregulation of pathogenic T_H_17 signature genes under DFMO@Ui‐Mn treatment (Figure 5F).

To validate whether STAT signaling was associated with the transcriptomic changes induced by DFMO@Ui‐Mn, we examined pathway‐related proteins at the post‐translational level. Because transcriptomic analysis highlighted Socs2 as a negative‐feedback regulator within the JAK–STAT module, and STAT3 and STAT5 exert opposing effects on T_H_17 programs [28], we next examined both STAT3 and STAT5 signaling. Immunoblotting showed reduced STAT3 phosphorylation, partial restoration of STAT5 phosphorylation, and increased SOCS2 abundance in the DFMO@Ui‐Mn group relative to T_H_17, without obvious changes in total STAT3 or STAT5 (Figure 5G,H). Immunofluorescence imaging further showed weaker p‐STAT3 and stronger p‐STAT5 signals under DFMO@Ui‐Mn treatment (Figure 5I). Together, these results indicate that DFMO@Ui‐Mn treatment is associated with altered STAT3/STAT5 signaling accompanied by SOCS2 upregulation, in parallel with reduced T_H_17‐associated transcriptional features.

### 2.6 DFMO@Ui‐Mn Reduces Alveolar Bone Loss in Ligature‐induced Periodontitis

**FIGURE 6:**
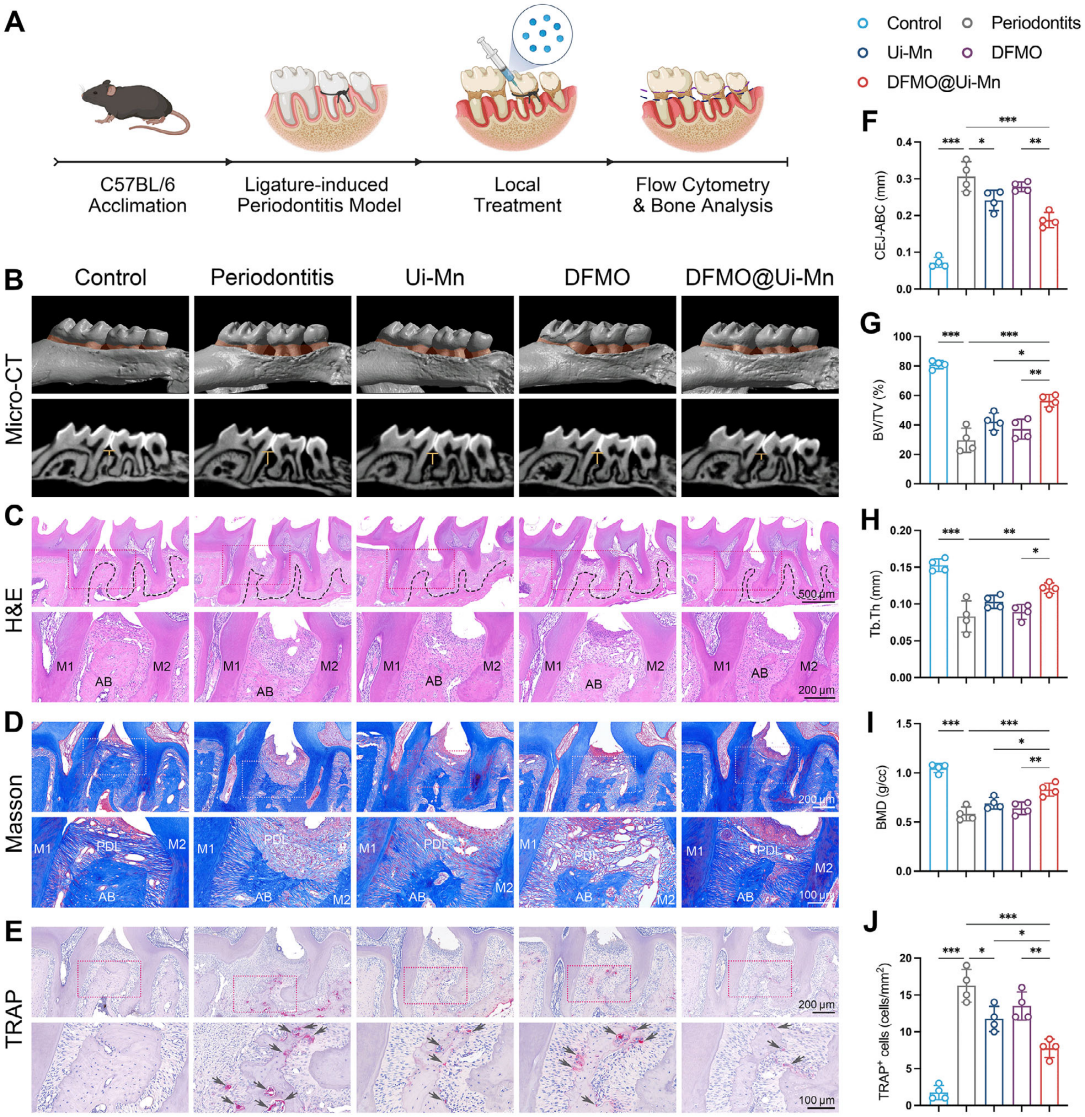
DFMO@Ui‐Mn promotes alveolar bone repair in ligature‐induced periodontitis. (A) Workflow of the mouse periodontitis model and treatment/analysis schedule. (B) Representative micro‐CT 3D reconstructions and sagittal sections of maxillae; colored markers highlight areas of alveolar bone loss. (C) Representative H&E staining and (D) Masson's trichrome staining of periodontal tissues (upper panels) with magnified views of the boxed regions (lower panels). Dashed lines outline the alveolar bone contour. PDL, periodontal ligament; AB, alveolar bone; M1, the first molar; M2, the second molar. (E) Representative TRAP staining in the interproximal region between M1 and M2; arrows denote TRAP‐positive cells. (F) Quantification of CEJ‐ABC distance at the second molar. (G) Bone volume fraction (BV/TV). (H) Trabecular thickness (Tb.Th). (I) Bone mineral density (BMD). (J) Quantification of TRAP‐positive cells in the interproximal region (*n* = 4). Data are presented as mean ± standard deviation and were analyzed using one‐way ANOVA followed by Tukey's multiple‐comparisons test. *p < 0.05, **p < 0.01, ***p < 0.001.

A ligature‐induced periodontitis model was established in C57BL/6 mice, followed by intragingival administration of DFMO@Ui‐Mn according to the experimental scheme (Figure 6A). Systemic biosafety was first examined, and histological inspection of major organs showed no noticeable pathological alterations (Figure S9). Alveolar bone destruction was then evaluated by micro‐CT with three‐dimensional reconstruction (Figure 6B). Marked bone loss and furcation‐region resorption were evident in the periodontitis group receiving saline injections. DFMO treatment showed a modest benefit, Ui‐Mn produced a clearer improvement, and DFMO@Ui‐Mn showed the best overall outcome. This was supported by a reduced distance from the cementoenamel junction to the alveolar bone crest (CEJ‐ABC) and improved bone microarchitectural parameters, including bone volume fraction (BV/TV), trabecular thickness (Tb.Th), and bone mineral density (BMD) (Figure 6F–I).

Histological analyses further supported tissue preservation after DFMO@Ui‐Mn treatment. Hematoxylin and eosin (H&E) staining and Masson's trichrome staining revealed disrupted alveolar bone morphology and disorganized periodontal ligament fibers in the periodontitis group. These pathological changes were alleviated in the DFMO@Ui‐Mn group, showing better maintenance of the alveolar crest contour and more organized collagen fiber arrangement (Figure 6C,D). Because osteoclast accumulation is a key driver of inflammatory bone resorption, tartrate‐resistant acid phosphatase (TRAP) staining was used to assess osteoclast presence in the interproximal region. An increased presence of TRAP‐positive cells was observed in the periodontitis group, whereas TRAP‐positive cells were reduced in the DFMO@Ui‐Mn group (Figure 6E), which was further supported by quantitative analysis (Figure 6J).

### 2.7 DFMO@Ui‐Mn Attenuates T_H_17 Skewing, Lowers the T_H_17/Treg Ratio and Suppresses Local Inflammatory Signals

**FIGURE 7:**
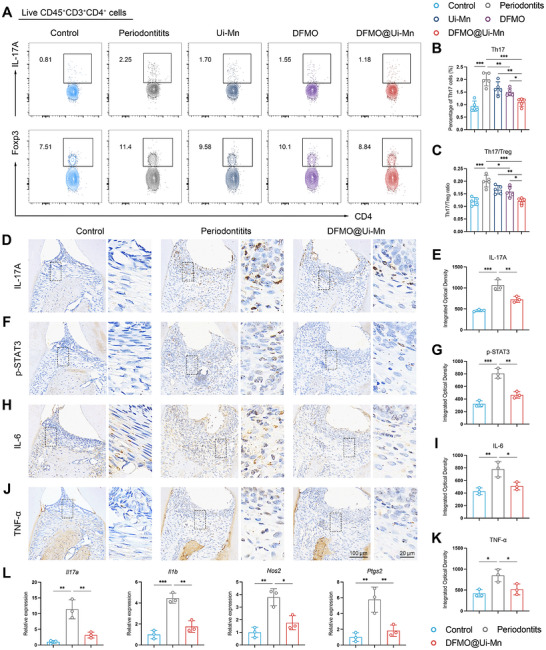
DFMO@Ui‐Mn lowers T_H_17/Treg ratio and suppresses inflammatory signaling in vivo. (A) Representative flow cytometry plots of IL‐17A^+^ T_H_17 cells and Foxp3^+^ Treg cells gated on live CD45^+^CD3^+^CD4^+^ lymphocytes from cervical lymph nodes in the indicated groups. (B) Quantification of T_H_17 frequency (*n* = 5). (C) Quantification of the T_H_17/Treg ratio (*n* = 5). (D) Representative IHC staining of IL‐17A in periodontal tissues and (E) corresponding integrated optical density (IOD) quantification (*n* = 3). (F) Representative IHC staining of p‐STAT3 and (G) IOD quantification (*n* = 3). (H) Representative IHC staining of IL‐6 and (I) IOD quantification (*n* = 3). (J) Representative IHC staining of TNF‐α and (K) IOD quantification (*n* = 3). (L) RT‐qPCR analysis of inflammatory gene expression in gingival tissues (*Il17a*, *Il1b*, *Nos2*, and *Ptgs2*) (*n* = 3). Data are presented as mean ± standard deviation and were analyzed using one‐way ANOVA followed by Tukey's multiple‐comparisons test. *p < 0.05, **p < 0.01, ***p < 0.001.

To assess the in vivo immunoregulatory and anti‐inflammatory effects of DFMO@Ui‐Mn, we examined T_H_17 responses and the T_H_17/Treg ratio in cervical lymph nodes, the major draining lymphoid sites for periodontal tissues. Ligature‐induced periodontitis significantly increased the frequency of IL‐17A^+^ T_H_17 cells and elevated the T_H_17/Treg ratio compared to controls (Figure 7A–C). Free DFMO and Ui‐Mn each partially mitigated these changes, whereas DFMO@Ui‐Mn produced the largest decrease in T_H_17 frequency and T_H_17/Treg ratio (Figure 7B,C). Corroborating these immunological changes, immunohistochemical staining of periodontal tissues showed that the elevated levels of IL‐17A and p‐STAT3 observed in periodontitis were markedly attenuated following DFMO@Ui‐Mn treatment (Figure 7D–G). Given the critical role of T_H_17 responses in amplifying local inflammation, we further examined inflammatory mediators. The inflammatory microenvironment was significantly ameliorated, as evidenced by reduced positive staining for IL‐6 and TNF‐α (Figure 7H–K). Furthermore, RT‐qPCR analysis of gingival tissues confirmed that key proinflammatory genes (*Il17a*, *Il1b*, *Nos2*, and *Ptgs2*) were upregulated in periodontitis but downregulated upon DFMO@Ui‐Mn administration (Figure 7L). Collectively, these findings indicate that DFMO@Ui‐Mn attenuates periodontitis‐associated T_H_17 skewing and is accompanied by reduced p‐STAT3 staining and lower local inflammatory readouts in vivo.

## 3 Discussion

The periodontal immune microenvironment is increasingly recognized as a major determinant of alveolar bone remodeling. Single‐cell transcriptomic analyses have demonstrated enhanced infiltration of neutrophils, plasma cells, and T_H_17 cells in periodontitis [29, 30]. In particular, IL‐17 has emerged as a critical mediator of alveolar bone loss [31], with T_H_17 cells serving as the primary cellular source of this cytokine in periodontal tissues [10]. Consistent with these reports, our data showed that *Il17a* was the most prominently upregulated helper T cell‐associated marker in diseased gingiva, accompanied by elevated IL‐17A expression and an increased T_H_17 frequency in cervical lymph nodes. These observations support T_H_17‐associated responses as a therapeutically relevant component of inflammatory bone loss [10, 32]. Metabolic reprogramming is a major determinant of T cell lineage commitment and functional specialization. In the present study, in vitro T_H_17 polarization was accompanied by enrichment of arginine‐related metabolic signatures and increased expression of polyamine pathway genes. Previous studies have reported elevated arginase and ODC expression in established periodontal lesions [33]. Our data therefore place polyamine‐related metabolism in closer association with T_H_17 responses in periodontitis and suggest that ODC inhibition may be a useful avenue for modulating this inflammatory program. Notably, polyamine metabolism may also contribute to oxidative stress, as polyamine catabolism can generate ROS as metabolic byproducts [34]. Accordingly, dysregulated polyamine metabolism in periodontitis may contribute both to T_H_17‐associated inflammation and to redox imbalance within the local tissue environment.

Based on the observed association between T_H_17 responses, polyamine‐related metabolic programs, and persistent oxidative stress in periodontitis, we constructed DFMO@Ui‐Mn to jointly modulate polyamine metabolism and redox imbalance. This design was inspired by the metabolic profiling results and added an immunometabolic dimension to the antioxidant nanozyme strategy. DFMO@Ui‐Mn suppressed T_H_17 polarization more effectively than either DFMO or Ui‐Mn alone. The stronger effect of DFMO‐containing groups is consistent with the possibility that polyamine metabolism contributes to T_H_17 polarization in this system. At the same time, DFMO@Ui‐Mn outperformed DFMO alone, suggesting an added contribution from the Ui‐Mn component. Ui‐Mn by itself exerted only a modest inhibitory effect on T_H_17 differentiation, in line with previous evidence that ROS scavenging can restrain T_H_17 programs; for instance, N‐acetylcysteine, a classical antioxidant, has been reported to reduce T_H_17 differentiation [35, 36]. Consequently, these data suggest that combining DFMO‐mediated polyamine restriction with Ui‐Mn–based ROS buffering may dampen T_H_17‐associated responses more effectively than either intervention alone.

Recent osteoimmunomodulatory studies have shown that biomaterials can reshape adaptive immune responses and T cell–dependent paracrine signaling to create a more pro‐osteogenic secretome that supports bone regeneration [37, 38]. In line with this, our results showed that T_H_17‐CM markedly suppresses osteogenic programs in BMSCs. This aligns with previous reports that IL‐17A disrupts key osteogenic pathways such as Wnt signaling, inhibits osteogenic differentiation [39], and compromises the regenerative functions of periodontal cells [40]. Notably, conditioned medium derived from DFMO@Ui‐Mn‐treated T cells partially restored osteogenic readouts suppressed by T_H_17‐CM, suggesting that modulation of the T cell secretome contributes to the observed osteogenic benefit. This interpretation is further supported by previous evidence showing that attenuation of IL‐17‐dominant T cell inflammatory signaling helps preserve osteogenic activity and matrix mineralization [41].

In vivo, Ui‐Mn alone showed measurable protection against alveolar bone loss, aligning with previous findings that Mn‐containing UiO‐66 platforms mitigate periodontitis partly through antioxidative mechanisms [42]. This therapeutic benefit was more pronounced in the tissue setting than in isolated T_H_17‐polarization assays, which may reflect the broader effects of ROS scavenging across stromal cells, innate immune cells, and osteoclast lineage cells [43, 44]. Although DFMO@Ui‐Mn markedly reduced TRAP‐positive osteoclasts in the lesion area, this effect should be interpreted in the context of the remodeled inflammatory microenvironment. Given that osteoclast accumulation in periodontitis is strongly shaped by T_H_17/IL‐17‐RANKL signaling [45] and local oxidative stress [46], the reduction in TRAP‐positive cells may at least partly reflect improvement of the local osteoimmune microenvironment. The superior efficacy of DFMO@Ui‐Mn supports the added value of combining ROS control with DFMO‐based modulation of adaptive immune responses, particularly in restraining T_H_17‐associated responses. This enhanced effect is also consistent with a potential benefit of local co‐localization of ROS‐scavenging activity and DFMO delivery, although tissue retention was not directly measured here. In a related study, locally injected UiO‐66‐based MOF nanoparticles remained detectable in periodontal tissues for at least 48 h after administration [47]. At the molecular level, the combined therapeutic effect may be associated with coordinated regulation of redox homeostasis and immunometabolic modulation. Oxidative stress has been reported to promote STAT3 tyrosine phosphorylation and nuclear translocation [48]. Therefore, the ROS‐scavenging activity of Ui‐Mn may contribute to the attenuation of ROS‐dependent STAT3 activation by restoring redox homeostasis. Together with DFMO‐mediated interference with polyamine metabolism, this redox‐regulatory effect may help shift the STAT3/STAT5 signaling balance away from T_H_17‐promoting activation. Overall, these results suggest that combining redox control with immunometabolic intervention can improve inflammatory and bone‐loss outcomes more than either approach alone in this model.

Several limitations should be noted. First, the current data support the involvement of ODC‐dependent polyamine metabolism in T_H_17 polarization, but do not establish cell‐type‐specific target engagement in vivo. Second, the signaling analyses link DFMO@Ui‐Mn treatment to increased SOCS2 expression and altered STAT3/STAT5 signaling, although causal mediation has not been tested. Future studies using SOCS2 knockdown will be needed to determine whether SOCS2‐dependent feedback contributes to DFMO@Ui‐Mn–mediated T_H_17 suppression. In addition, the current platform lacks cell‐type specificity; future iterations may benefit from targeting motifs that enrich delivery to pathogenic cell populations [49, 50].

## 4 Conclusion

In summary, this study identified a pronounced T_H_17‐skewed CD4^+^ T cell response in experimental periodontitis and observed polyamine pathway activation during T_H_17 differentiation as a candidate immunometabolic feature associated with this response. Based on this insight, we developed the DFMO@Ui‐Mn nanozyme platform, which combines ROS buffering with DFMO delivery to modulate polyamine metabolism. Rather than relying on a single‐function antioxidant approach, the present work suggests that concurrent control of oxidative stress and T_H_17‐associated inflammatory programs can improve the periodontal tissue environment. In our model, DFMO@Ui‐Mn reduced inflammatory readouts, lowered the T_H_17/Treg ratio, improved osteogenic readouts in a conditioned‐medium system, and promoted alveolar bone repair. This strategy supports further exploration of locally delivered immunometabolic therapy in periodontitis and potentially other inflammatory bone‐loss conditions in which redox imbalance and immune dysfunction coexist. More broadly, our findings suggest that combining control of tissue‐damaging redox stress with modulation of pathogenic immune programs may improve conditions for tissue repair.

## 5 Experimental Section

### 5.1 Preparation of DFMO@UiO‐66(Ce)‐Mn

The synthesis was adapted from the literature [51]. F127 (100 mg, 0.00833 mmol) was dissolved in deionized water (6 mL), followed by addition of acetic acid (HAc, 0.05 mL, 0.85 mmol), toluene (400 µL), and NaClO_4_·H_2_O (409 mg, 2.92 mmol). The mixture was stirred in a constant‐temperature water bath at 11°C for 18 h to form a homogeneous microemulsion. Terephthalic acid (BDC, 166 mg, 1 mmol), MnTCPP (42.2 mg, 0.05 mmol), and (NH_4_)_2_Ce(NO_3_)_6_ (548 mg, 1 mmol) were then added and the reaction was continued at 11°C for 4 h. The solids were collected by centrifugation and washed twice with water, followed by two DMSO washes to remove unreacted ligands. The product was then washed with ethanol and soaked in fresh ethanol at 60°C for 2 days with daily solvent exchange, followed by vacuum drying at 80°C for 24 h to obtain UiO‐66(Ce)‐Mn.

Eflornithine hydrochloride hydrate (DFMO hydrochloride hydrate) loading was performed post‐synthesis by dispersing UiO‐66(Ce)‐Mn (5.0 mg) in 5 mL aqueous DFMO solution (10.0 mg) and stirring overnight at 4°C. The DFMO‐loaded solids were collected by centrifugation and washed three times with PBS to remove unbound DFMO, yielding DFMO@UiO‐66(Ce)‐Mn.

### 5.2 Characterization of DFMO@UiO‐66(Ce)‐Mn

SEM images were acquired using a Regulus 8100 field‐emission scanning electron microscope (Hitachi). TEM imaging and energy‐dispersive X‐ray spectroscopy (EDS) elemental mapping were performed using a Talos F200X G2 (FEI). Particle hydrodynamic size was measured by dynamic light scattering using a Zetasizer Nano ZS90 (Malvern). XRD patterns were recorded on a SmartLab SE diffractometer (Rigaku) with Cu Kα radiation at a scan rate of 5°/min. Raman spectra were recorded using a LabRAM Odyssey Raman microscope (HORIBA). XPS characterization was performed on a K‐Alpha XPS system (Thermo Scientific). N_2_ sorption isotherms were acquired at 77 K on an ASAP 2460 analyzer (Micromeritics), after degassing all samples under vacuum at 100°C for 12 h. DFMO in the supernatants and release samples was quantified by HPLC using a Boston Green ODS‐AQ column. The mass of loaded DFMO was calculated by subtracting the mass of unbound DFMO detected in the supernatants from the mass of DFMO initially added. Encapsulation efficiency (%) = mass of loaded DFMO / mass of DFMO initially added × 100%. Drug loading content (%) = mass of loaded DFMO / mass of DFMO‐loaded nanoparticles × 100%. The removal of H_2_O_2_ was quantified using an H_2_O_2_ assay kit (Beyotime). Dissolved O_2_ levels were monitored using a dissolved oxygen electrode.

### 5.3 In Vitro Biocompatibility Assessment

Primary CD4^+^ T cells were incubated with DFMO@Ui‐Mn. Cell viability was evaluated using a Cell Counting Kit‐8 (CCK‐8, Dojindo) assay. At the end of the incubation, CCK‐8 reagent was added at 10% of the culture volume, and the absorbance at 450 nm was measured using a microplate reader (BioTek). Cell viability was further assessed with a Zombie NIR Fixable Viability Kit (BioLegend). Apoptosis was analyzed using an Annexin V/PI kit (Beyotime). Flow cytometry data were acquired on a NovoCyte flow cytometer (Agilent) and analyzed with FlowJo software (BD Biosciences).

### 5.4 Naïve CD4^+^ T cell isolation and T_H_17 polarization

Naïve CD4^+^ T cells were magnetically isolated from spleens of 8‐week‐old male C57BL/6 mice using a Naïve CD4^+^ T Cell Isolation Kit (StemCell Technologies). Cells were seeded in 24‐well plates and activated with plate‐bound anti‐mouse CD3 (5 µg/mL; BioLegend) and soluble anti‐mouse CD28 (5 µg/mL; BioLegend). Primary CD4^+^ T cells were cultured in IMDM (Gibco) supplemented with 10% FBS (Excell), 1% penicillin‐streptomycin (Gibco), and 55 µM β‐mercaptoethanol (Gibco). T_H_17 differentiation was induced with rmIL‐6 (50 ng/mL; PeproTech), rmTGF‐β1 (1 ng/mL; Sino Biological), rmIL‐23 (10 ng/mL; Sino Biological), anti‐mouse IFN‐γ (10 µg/mL; BioLegend), anti‐mouse IL‐4 (10 µg/mL; BioLegend), and rmIL‐1β (10 ng/mL; PeproTech). Putrescine (Aladdin), DFMO (MCE), UiO‐66(Ce)‐Mn, or DFMO@UiO‐66(Ce)‐Mn were added to the T_H_17 induction medium as indicated.

### 5.5 Flow Cytometry

Single‐cell suspensions from cervical lymph nodes were prepared by mechanical dissociation through a 70‐µm strainer (Biosharp) using the rubber end of a 10‐mL syringe plunger. For cells from in vivo tissues or CD4^+^ T cells after in vitro culture, cells were resuspended in IMDM and stimulated with Cell Activation Cocktail with Brefeldin A (BioLegend) for 5 h at 37°C. Samples were Fc‐blocked with TruStain FcX (BioLegend), stained with Zombie NIR Fixable Viability Kit (BioLegend), and then surface‐stained for CD4 (in vitro) or CD45/CD3/CD4 (in vivo). Cells were fixed and permeabilized using the fixation/intracellular staining perm wash set (BioLegend) followed by intracellular staining with anti‐IL‐17A. For samples requiring Foxp3 detection, fixation/permeabilization and staining were performed with the Foxp3/Transcription Factor Staining Buffer Set (eBioscience) according to the manufacturer's instructions. Antibodies are listed in Table S2. The gating strategy is shown in Figure S10. Data were acquired on a NovoCyte flow cytometer (Agilent) and analyzed with FlowJo.

### 5.6 Enzyme‐Linked Immunosorbent Assay (ELISA)

Supernatants from CD4^+^ T cell cultures were collected after centrifugation. IL‐17A levels were measured using a mouse IL‐17A Precoated ELISA kit (Dakewe) according to the manufacturer's instructions.

### 5.7 Real‐Time Quantitative Polymerase Chain Reaction (RT‐qPCR)

Total RNA was extracted with an RNA extraction kit (Esunbio), followed by reverse transcription with a cDNA synthesis kit (Takara). RT‐qPCR was performed with SYBR Green Master Mix (Takara) on a LightCycler 480 system (Roche). Relative expression was calculated by the 2^−ΔΔCt^ method, normalized to *Actb*. Primer sequences are listed in Table S3.

### 5.8 BMSC Isolation and Culture

Male C57BL/6 mice (3 weeks) were euthanized according to institutional guidelines. Femurs and tibias were aseptically harvested, cleared of soft tissue, and bone marrow was flushed with α‐MEM (Gibco) containing 10% FBS (Excell) and 1% penicillin‐streptomycin (Gibco). Cells were plated in 10‐cm dishes and cultured at 37°C, 5% CO_2_. After 24 h, non‐adherent cells were removed, and fresh medium was added; medium was then changed every 2–3 days. Adherent cells were detached with 0.05% trypsin‐EDTA (Gibco) at 80%–90% confluence.

### 5.9 Osteogenic Evaluation

CD4^+^ T cell‐conditioned medium (CM) was prepared from 3‐day culture supernatants, clarified by centrifugation, and mixed with α‐MEM at 1:2 (v/v). BMSCs were assessed for osteogenic gene/protein expression by RT‐qPCR, immunoblotting, and immunofluorescence. After 7 days of culture, ALP staining was performed using the NBT/BCIP kit (Beyotime). After 14 days, calcium deposition was evaluated with the Alizarin Red S kit (OriCell).

### 5.10 Immunoblotting

Cells were rinsed with PBS and lysed in RIPA buffer containing phosphatase and protease inhibitors (Signalway Antibody). Lysates were mixed with loading buffer and denatured at 95°C for 10 min. Proteins were separated by SDS‐PAGE and transferred to PVDF membranes (Millipore). Membranes were blocked with fast blocking buffer (Epizyme), incubated with primary antibodies overnight at 4°C, then with HRP‐conjugated goat anti‐mouse or anti‐rabbit secondary antibodies (Proteintech) for 1 h at room temperature.

### 5.11 Immunofluorescence

Cells were rinsed with PBS, fixed with 4% paraformaldehyde for 20 min, and permeabilized with 0.25% Triton X‐100 for 10 min. After blocking with fast blocking buffer (Beyotime) for 20 min, cells were incubated with primary antibodies overnight at 4°C and then with secondary antibodies for 1 h at room temperature. F‐actin was stained with Alexa Fluor 488 Phalloidin (Invitrogen); nuclei were counterstained with DAPI (Solarbio). Images were acquired using an inverted fluorescence microscope (Leica).

### 5.12 RNA sequencing and Data Analysis

Total RNA was extracted using the TRIzol method. The cDNA libraries were sequenced on the Illumina sequencing platform by Metware Biotechnology to yield 150‐bp paired‐end reads. Gene expression was quantified with featureCounts, and FPKM values were computed per gene. Differential expression was assessed with DESeq2 with Benjamini‐Hochberg correction; adjusted *p* values and log_2_ fold change served as significance thresholds. Functional enrichment was performed by hypergeometric testing for GO terms and KEGG pathways. Gene set enrichment analysis (GSEA) was conducted using the clusterProfiler R package.

### 5.13 Untargeted Metabolomics Analysis

Cell pellets were extracted separately for hydrophilic and hydrophobic metabolites. Hydrophilic metabolites were extracted with methanol/water (4:1, v/v) containing internal standards by vortexing, freeze‐thaw cycles and centrifugation at 4°C, and the supernatant was used for LC‐MS analysis. Hydrophobic metabolites were extracted with MTBE/methanol (3:1, v/v) containing internal standards, phase‐separated with water, and the upper organic phase was collected, dried and reconstituted in acetonitrile/isopropanol (1:1, v/v). Polar and lipid extracts were analyzed on an ExionLC AD UPLC system coupled to a QTRAP 6500+ mass spectrometer (Sciex) using reversed‐phase and C30 columns under water/acetonitrile‐ and acetonitrile/isopropanol‐based gradients containing formic acid and ammonium salts. Mass spectra were acquired in positive and negative electrospray ionization modes. Peak data were log_2_‐transformed, scaled, and subjected to PCA and OPLS‐DA in R (MetaboAnalystR), with model robustness evaluated by 200‐permutation testing. Differential metabolites were defined as those with VIP > 1 in the OPLS‐DA model and Student's *t*‐test *p* < 0.05.

### 5.14 Targeted Metabolomics Analysis

Targeted metabolites were quantified by MetWare using an AB Sciex QTRAP 6500 LC‐MS/MS platform. Samples were thawed on ice and extracted with precooled 80% methanol/water, followed by vortexing, freeze‐thaw cycles, and centrifugation; the supernatant was further clarified and used for LC‐MS analysis. For selected metabolites, aliquots of the extract were derivatized with 3‐nitrophenylhydrazine/EDC and diluted before injection. Chromatographic separation was performed on reversed‐phase and amide UPLC columns under optimized water/acetonitrile gradients containing formic acid and ammonium salts. The mass spectrometer was operated in positive and negative electrospray ionization modes, and data were acquired in multiple reaction monitoring (MRM) mode with compound‐specific transitions.

### 5.15 Mice and Periodontitis Model

All procedures were approved by the Institutional Animal Care and Use Committee of Shanghai Ninth People's Hospital Affiliated to Shanghai Jiao Tong University School of Medicine (Approval No. SH9H‐2025‐A1703‐1). Male C57BL/6 mice were purchased from GemPharmatech Corporation and were maintained under pathogen‐free conditions on a 12‐h light/dark cycle with ad libitum access to food and water. Mice were acclimated for 1 week before experiments.

Ligature‐induced periodontitis was established in mice by creating a local biofilm‐retentive microenvironment around the maxillary second molars, thereby inducing alveolar bone loss [52]. Briefly, 8‐week‐old male C57BL/6 mice were anesthetized by intraperitoneal injection of a combined anesthetic regimen consisting of Shutai‐50 and dexmedetomidine hydrochloride. 6‐0 silk ligatures were tied around the maxillary second molar at both sides. One week after ligature insertion, mice received local injections of saline, DFMO, UiO‐66(Ce)‐Mn, or DFMO@UiO‐66(Ce)‐Mn twice weekly until sacrifice. For each administration, 8 µL of the corresponding formulation was injected around the ligated maxillary second molar using a microsyringe and evenly distributed across four gingival sites, including the mesiobuccal, distobuccal, mesiopalatal, and distopalatal regions. Mice were euthanized by CO_2_ inhalation. Cervical lymph nodes were harvested and processed into single‐cell suspensions as described above. Gingival tissues were collected on ice, homogenized in RNA lysis buffer using a tissue grinder, and stored at ‐80°C for subsequent RNA extraction. Maxillae and major organs were collected and fixed in 4% paraformaldehyde.

### 5.16 Micro‐CT and Immunohistology

Maxillae were scanned on a micro‐CT system (SkyScan 1276, Bruker) with the following settings: 60 kV source voltage, 200 µA source current, and 6.55 µm voxel size. Data were reconstructed with NRecon, rendered in 3D with CTvox, and analyzed using CTAn (Bruker). For histology, maxillae were decalcified, dehydrated, paraffin‐embedded, and sectioned at 4 µm, then stained with H&E, Masson, and TRAP kits (Servicebio) according to the manufacturer's instructions.

For immunohistology, paraffin sections were dewaxed, subjected to citrate antigen retrieval, and processed with an IHC kit (ZSGB‐bio) according to the manufacturer's instructions. Endogenous peroxidase was blocked with the kit blocker, followed by incubation with primary antibodies overnight at 4°C, secondary antibody incubation for 20 min at 37°C, DAB development, and hematoxylin counterstaining. For immunohistochemical quantification, predefined anatomical regions of interest (ROIs) were selected from the gingival tissue in the interdental region between the first and second molars. Images were acquired under identical settings, and the integrated optical density (IOD) within each ROI was measured using ImageJ software. All image analyses were performed in a blinded manner.

### 5.17 Statistical Analysis

All quantitative data were presented as the mean ± standard deviation (SD). Unless otherwise specified, data were analyzed without additional transformation. The sample size (n), representing biological replicates or independent experiments, was specified in the corresponding figure legends. Statistical analyses were performed using GraphPad Prism 8.0. Comparisons between two groups were performed using an unpaired two‐tailed Student's *t*‐test. Comparisons among more than two groups were performed using one‐way analysis of variance (one‐way ANOVA), followed by Tukey's multiple‐comparisons test. Statistical significance was indicated as *p < 0.05, **p < 0.01, and ***p < 0.001.

## Author Contributions

C.Z. and T.L.: Conceptualization, Investigation, Formal analysis, Visualization, Funding acquisition, Project administration, Writing – original draft. Y.J.: Methodology, Investigation, Formal analysis, Funding acquisition. Y.L. and Q.L.: Methodology, Investigation, Data curation, Validation. R.F., L.Q., and X.T.: Investigation, Data curation, Validation. R.L. and X.Z.: Data curation. J.H.: Conceptualization, Supervision, Resources, Writing – review & editing. B.F. and L.X.: Conceptualization, Supervision, Funding acquisition, Writing – review & editing.

## Conflicts of Interest

The authors declare no conflicts of interests.

## Supporting information




**Supporting File**: advs77247‐sup‐0001‐SuppMat.pdf.

## Data Availability

The data that support the findings of this study are available from the corresponding author upon reasonable request.
