# Supplementary material for: TH17–Associated Polyamine Metabolism‐Guided Nanozyme Promotes Alveolar Bone Repair in Periodontitis
Source: Adv Sci (Weinh). 2026 Aug 18:e77247. Online ahead of print. doi: 10.1002/advs.77247 (PMC13482937; doi:10.1002/advs.77247)
Supplement: Supplementary file 1 [file ADVS-9999-e77247-s001.pdf]

Supporting Information

T_H17–Associated Polyamine Metabolism–Guided Nanozyme Promotes Alveolar Bone Repair in Periodontitis

Authors

Cheng Zhu[1†], Tiancheng Li[1†], Yu Jin[1], Yixin Li[1], Qicheng Liu[2], Runzhe Fang[1], Luyao Qu[1], Xinyue Tang[1], Ruomei Li[1], Xiaoyue Zhao[1], Jin Hao[1*], Bing Fang[1*], Lunguo Xia[1*]

† These authors contributed equally to this work.

This file includes:

Figure S1–S10

Table S1–S3

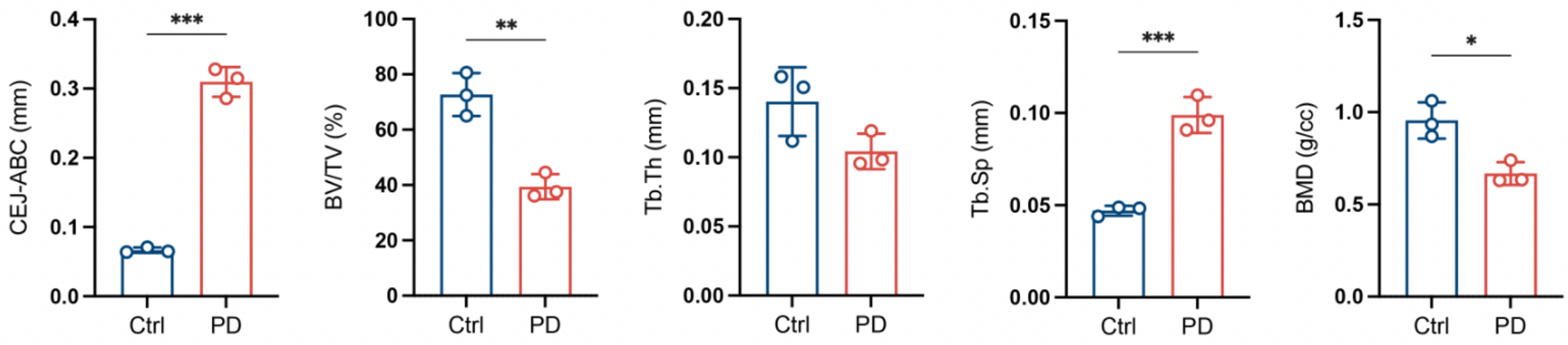

Figure S1. Quantitative micro-CT analysis of alveolar bone in control (Ctrl) and ligature-induced periodontitis (PD) groups. Cementoenamel junction to alveolar bone crest (CEJ–ABC) distance, bone volume fraction (BV/TV), trabecular thickness (Tb.Th), trabecular separation (Tb.Sp), and bone mineral density (BMD). n = 3. Data are presented as mean ± standard deviation and were analyzed using Student's t-test. *$P < 0.05$, **$P < 0.01$, ***$P < 0.001$.

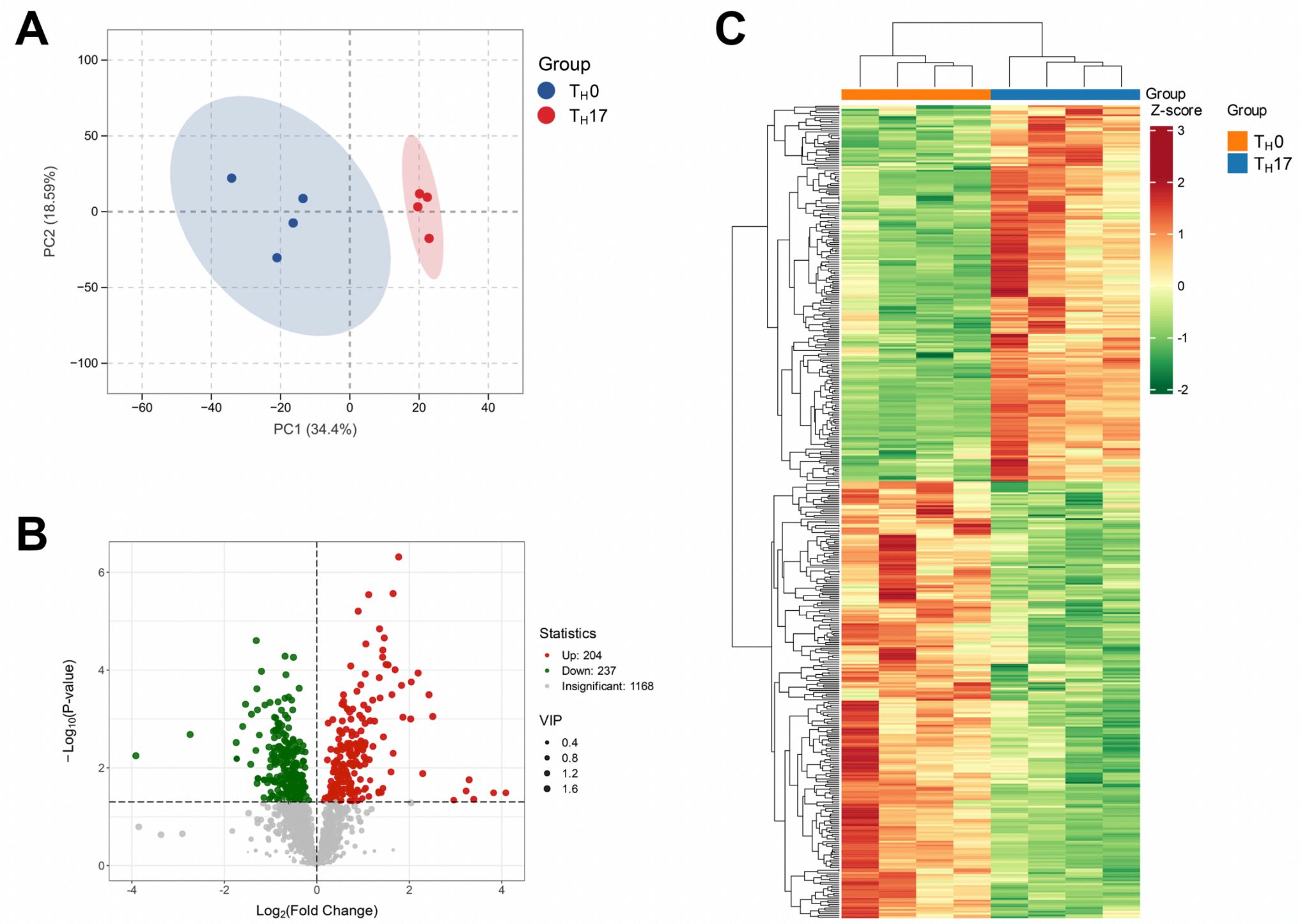

Figure S2. Untargeted metabolomic profiling of T_H17 and T_H0 cells. (A) Principal component analysis (PCA) score plot showing separation between T_H17 and T_H0 groups. (B) Volcano plot of differential metabolites between T_H17 and T_H0 cells; red and green dots indicate significantly increased and decreased metabolites, respectively. (C) Heatmap with hierarchical clustering of differentially abundant metabolites across groups (scaled as Z-scores). n = 4.

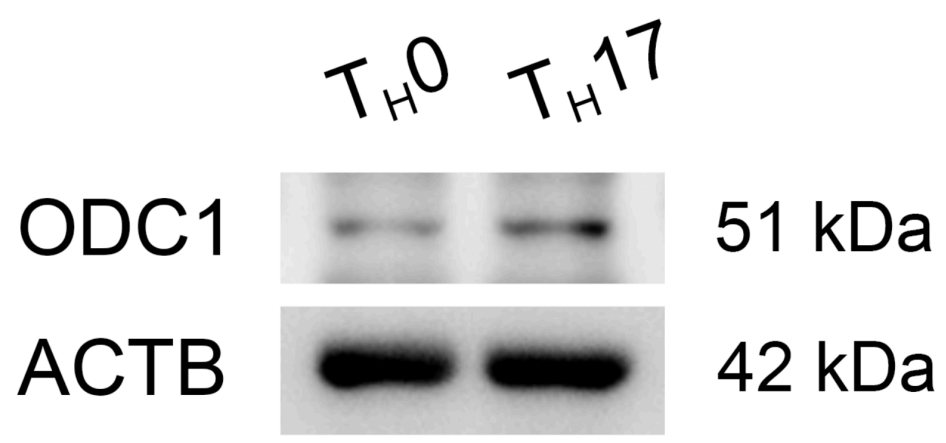

Figure S3. Representative Western blot images showing ODC1 protein expression in T_H0 and T_H17 cells. ACTB was used as the loading control.

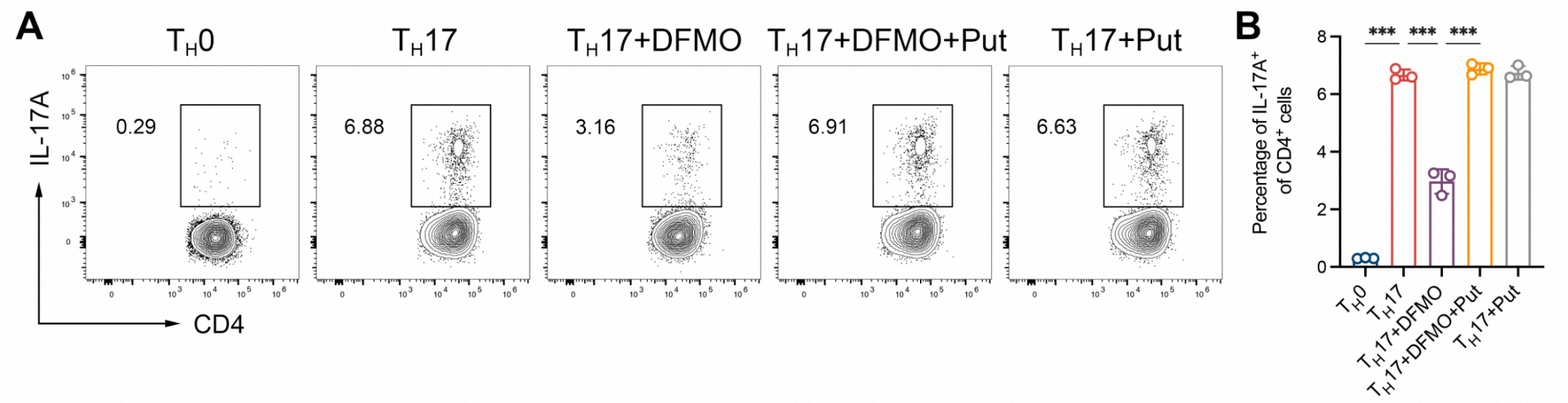

Figure S4. Putrescine supplementation rescues DFMO-mediated suppression of T_H17 polarization. (A) Representative flow cytometry plots showing IL-17A expression in *in vitro*-cultured $CD4^+$ T cells under the indicated conditions. DFMO was used at 100 μM, and putrescine (Put) was supplemented at 2.5 mM. (B) Quantification of IL-$17A^+$ cells among $CD4^+$ T cells. n = 3. Data are presented as mean ± standard deviation and were analyzed using one-way ANOVA followed by Tukey's multiple-comparisons test. ***$P < 0.001$.

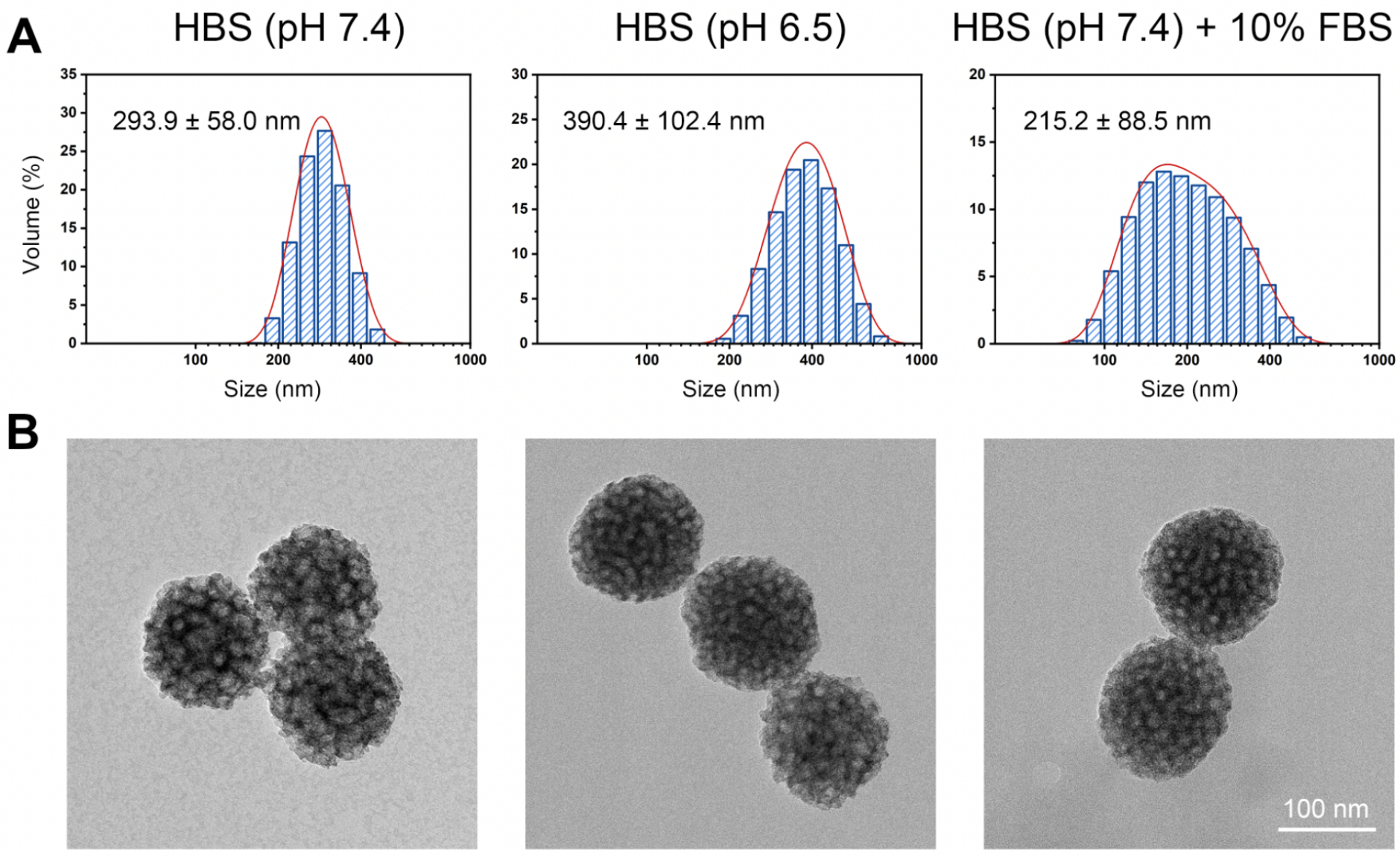

Figure S5. Morphological and hydrodynamic size characterization of DFMO@UiO-66(Ce)-Mn after incubation in different media. (A) DLS size distributions of DFMO@UiO-66(Ce)-Mn after incubation in HBS (pH 7.4), HBS (pH 6.5), or HBS (pH 7.4) containing 10% FBS for 3 days. The main hydrodynamic size peaks are indicated in each panel. (B) Representative TEM images showing the morphology of DFMO@UiO-66(Ce)-Mn after incubation in the indicated media.

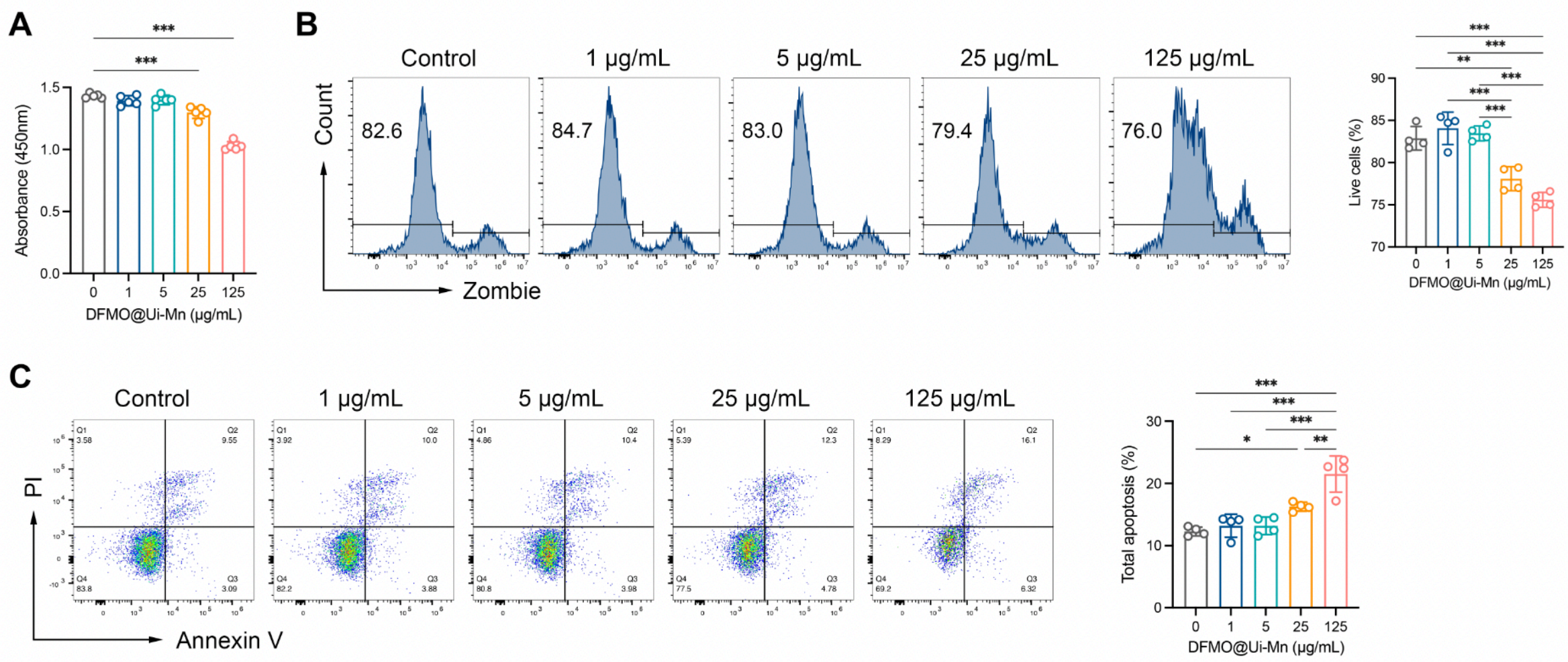

Figure S6. *In vitro* biocompatibility assessment of DFMO@Ui-Mn. (A) CCK-8 assay showing cell viability on day 3 after exposure to the indicated concentrations of DFMO@Ui-Mn (n = 5). (B) Representative flow-cytometry histograms and quantification of viable cells measured by Zombie viability dye on day 3 (n = 4). (C) Representative Annexin V/propidium iodide (PI) staining plots and quantification of total apoptotic cells (Annexin V$^+$) on day 3 (n = 4). Data are presented as mean ± standard deviation and were analyzed using one-way ANOVA followed by Tukey's multiple-comparisons test. *$P < 0.05$, **$P < 0.01$, and ***$P < 0.001$.

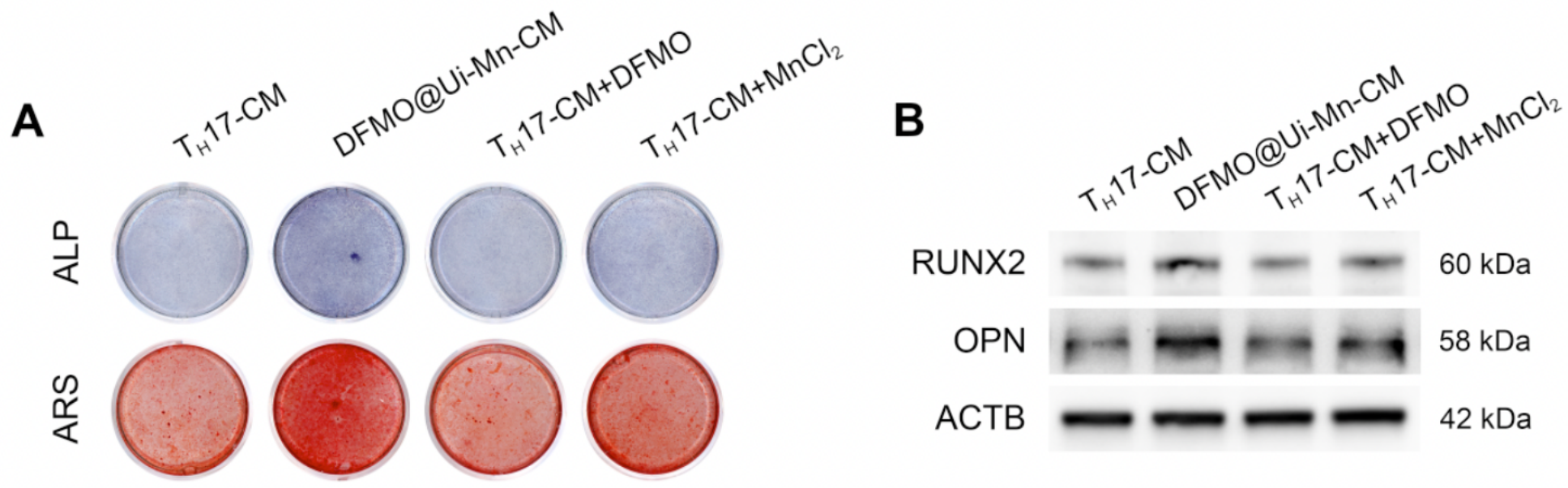

Figure S7. Osteogenic evaluation of BMSCs under T_H17-conditioned stimulation. (A) Representative alkaline phosphatase (ALP) and Alizarin Red S (ARS) staining. (B) Representative immunoblots of osteogenic markers RUNX2 and OPN, with ACTB as a loading control. T_H17-CM, BMSCs cultured with conditioned medium (CM) from T_H17 cells; DFMO@Ui-Mn-CM, BMSCs cultured with CM from T_H17 cells treated with DFMO@Ui-Mn; T_H17-CM + DFMO, BMSCs cultured with T_H17-CM supplemented with free DFMO; T_H17-CM + $MnCl_2$, BMSCs cultured with T_H17-CM supplemented with $MnCl_2$.

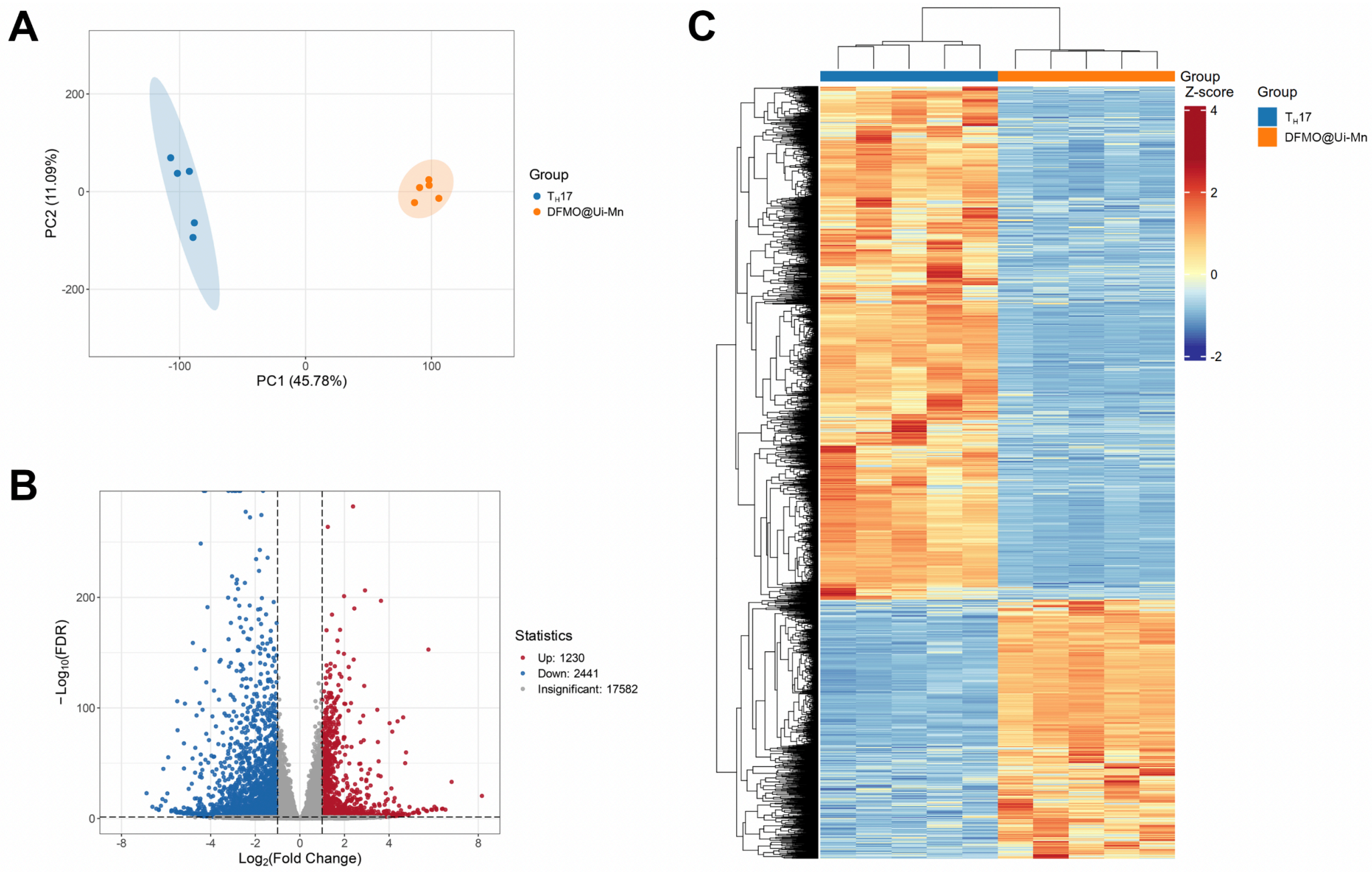

Figure S8. Transcriptomic profiling of T_H17 cells with or without DFMO@Ui-Mn treatment. (A) PCA score plot showing separation between T_H17 and DFMO@Ui-Mn-treated T_H17 groups. (B) Volcano plot of differentially expressed genes between DFMO@Ui-Mn and T_H17; red and blue dots indicate significantly upregulated and downregulated genes, respectively. (C) Heatmap with hierarchical clustering of DEGs across groups (scaled as Z-scores). n = 5.

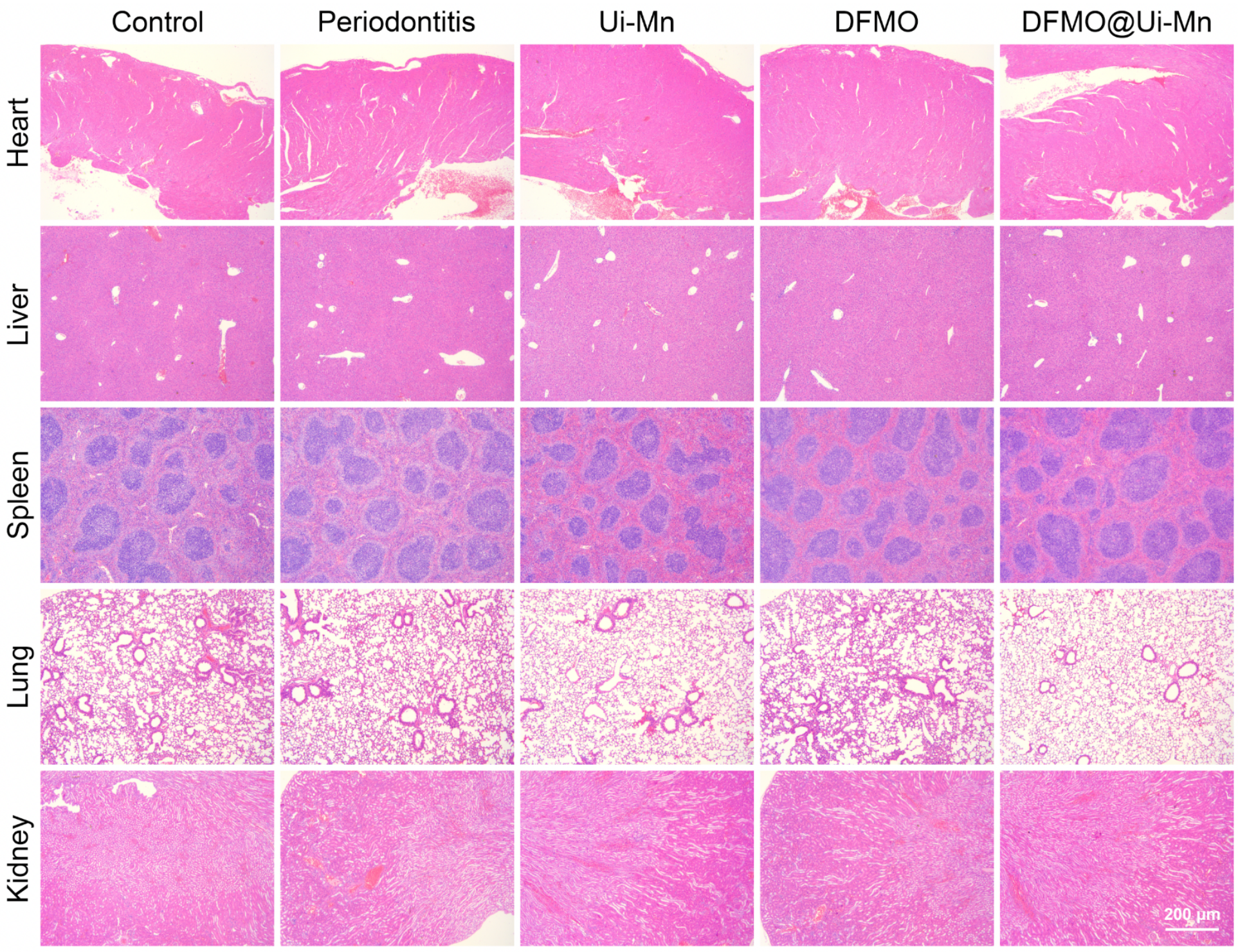

Figure S9. Histological assessment of major organs. Representative H&E staining images of the heart, liver, spleen, lung, and kidney from the indicated groups.

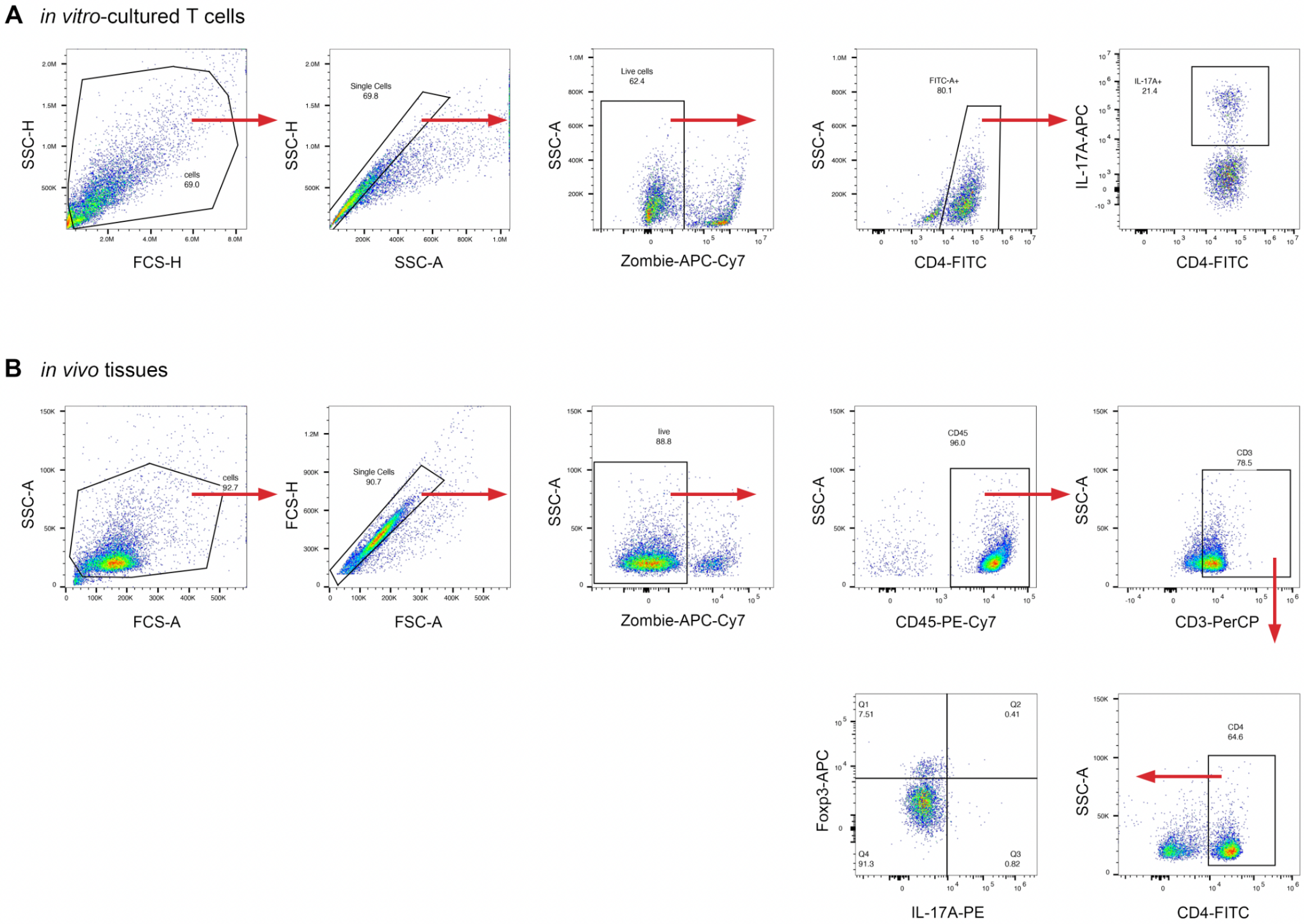

Figure S10. Flow cytometry gating strategy. (A) Representative gating scheme for T cells isolated and cultured *in vitro*. (B) Representative gating scheme for *in vivo* samples.

Table S1. Porosity parameters of UiO-66(Ce)-Mn before and after DFMO loading.

Sample	BET surface area (m^2/g)	Total pore volume (cm^3/g)	Calculated average pore diameter (nm)
UiO-66(Ce)-Mn	416.6	0.82	7.9
DFMO@UiO-66(Ce)-Mn	262.5	0.49	7.5

Table S2. List of antibodies

Antibody	Source	Identifier
FITC anti-mouse CD4	BioLegend	Cat#100509
PE/Cyanine7 anti-mouse CD45	BioLegend	Cat#103114
PerCP anti-mouse CD3ε	BioLegend	Cat#100326
PE anti-mouse IL-17A	BioLegend	Cat#506903
APC anti-mouse IL-17A	eBioScience	Cat#17-7177-81
Alexa Fluor® 647 anti-mouse/rat/human FOXP3	BioLegend	Cat#320014
ODC1 Monoclonal antibody	Proteintech	Cat#67336-1-Ig
Collagen I alpha 1 Rabbit pAb	ZenBio	Cat#501352
RUNX2 Recombinant Rabbit mAb	Huabio	Cat#ET1612-47
Osteopontin Polyclonal antibody	Proteintech	Cat#22952-1-AP
Stat3 (124H6) Mouse mAb	Cell Signaling Technology	Cat#9139
Phospho-Stat3 (Tyr705) (D3A7) Rabbit mAb	Cell Signaling Technology	Cat#9145
Stat5 (D2O6Y) Rabbit mAb	Cell Signaling Technology	Cat#94205
Phospho-Stat5 (Tyr694) (C11C5) Rabbit mAb	Cell Signaling Technology	Cat#9359
SOCS2 Antibody	Cell Signaling Technology	Cat#2779
Anti-IL-17 Rabbit pAb	Servicebio	Cat#GB11110-1-50
IL-6 Rabbit pAb	ZenBio	Cat#500286
TNF-alpha Monoclonal antibody	Proteintech	Cat#60291-1-Ig
Ultra-LEAF Purified anti-mouse CD3	BioLegend	Cat#100239
Ultra-LEAF Purified anti-mouse CD28	BioLegend	Cat#102115
Ultra-LEAF Purified anti-mouse IFN-γ Antibody	BioLegend	Cat#517905
Ultra-LEAF Purified anti-mouse IL-4 Antibody	BioLegend	Cat#504122

Table S3. RT-qPCR primer sequences

Gene	Forward	Reverse
Ifng	ATGAACGCTACACACTGCATC	CCATCCTTTTGCCAGTTCCTC
Il4	GGTCTCAACCCCCAGCTAGT	GCCGATGATCTCTCTCAAGTGAT
Il17a	TTTAACTCCCTTGGCGCAAAA	CTTTCCCTCCGCATTGACAC
Foxp3	CCCATCCCCAGGAGTCTTG	ACCATGACTAGGGGCACTGTA
Odc1	GACGAGTTTGACTGCCACATC	CGCAACATAGAACGCATCCTT
Sat1	GAGAACACCCCTTCTACCACT	GCCTCTGTAATCACTCATCACGA
Sms	GACCTTCAGAGTTACGACAGTG	GAACTATGGGTGGTAATCGCTTC
Srm	ACATCCTCGTCTTCCGCAGTA	GGCAGGTTGGCGATCATCT
Smox	TGGCCTGTAGTCGTGGAGT	GCATGGCCTAAAGAAACTGGTG
Paox	CTTCGGTGGTGTAGTGGAGC	TCCGATAATTCTTTCTCCCCCAG
Il17f	GAAGGCTGGGAACTGTCCTC	TGATGCAGCCTGAGTGTCTG
Il22	ATGAGTTTTTCCCTTATGGGGAC	GCTGGAAGTTGGACACCTCAA
Rorc	AGGCCATTCAGTATGTGGTGGAGT	TGTGTGGTTGTTGGCATTGTAGGC
Alp	GGTAGATTACGCTCACAACAAC	CAGGCACAGTGGTCAAGG
Col1a1	CACCCTCAAGAGCCTGAGTC	GTTCGGGCTGATGTACCAGT
Runx2	TACTTCGTCAGCATCCTATCAG	CAGCGTCAACACCATCATTC
Opn	GACGATGATGACGATGATGATG	CGACTGTAGGGACGATTGG
Il1b	GAAATGCCACCTTTTGACAGTG	TGGATGCTCTCATCAGGACAG
Nos2	GTTCTCAGCCCAACAATACAAGA	GTGGACGGGTCGATGTCAC
Ptgs2	TGCACTATGGTTACAAAAGCTGG	TCAGGAAGCTCCTTATTTCCCTT
